# Bergamot (*Citrus bergamia*) Supplementation and Cardiovascular Disease Risk Factors: A Systematic Review and Meta‐Analysis

**DOI:** 10.1002/fsn3.72357

**Published:** 2026-09-15

**Authors:** Mahla Chambari, Helia Mardani, Marjan Roshandel, Hossein Rafiei, Whitney Linsenmeyer, Maryam Jafari, Vaidehi Ulaganathan, Ali Jafari

**Affiliations:** ^1^ Department of Food Science and Nutrition, Faculty of Applied Sciences UCSI University Cheras, Wilayah Persekutuan Kuala Lumpur Malaysia; ^2^ Faculty of Medicine Navoi State University Navoi Uzbekistan; ^3^ Students' Scientific Research Center (SSRC) Tehran University of Medical Sciences Tehran Iran; ^4^ Master Student in Medical Surgical Nursing, Student Research Committee, Faculty of Nursing and Midwifery Mashhad University of Medical Sciences Mashhad Iran; ^5^ Department of Nutrition and Dietetics Saint Louis University Saint Louis Missouri USA; ^6^ Student Research Committee, School of Medicine, Anzali International Campus Guilan University of Medical Sciences Rasht Iran; ^7^ Student Research Committee, Department of Community Nutrition, Faculty of Nutrition Sciences and Food Technology, National Nutrition and Food Technology Research Institute Shahid Beheshti University of Medical Sciences Tehran Iran; ^8^ Systematic Review and Meta‐Analysis Expert Group (SRMEG) Universal Scientific Education and Research Network (USERN) Tehran Iran

**Keywords:** bergamot, *Citrus bergamia*, glycemic profile, lipid profile, meta‐analysis

## Abstract

Cardiovascular diseases remain the leading cause of global mortality. Bergamot ( *Citrus bergamia* ), a flavonoid‐rich nutraceutical, has shown potential cardiometabolic benefits, yet evidence from human trials remains inconsistent. This systematic review and dose–response meta‐analysis evaluated the effects of bergamot ( *Citrus bergamia* ) on cardiovascular disease risk factors in adults. We conducted a systematic review and meta‐analysis of randomized controlled trials following PRISMA guidelines (PROSPERO: CRD420251127505). PubMed, Web of Science, Scopus, Embase, and the Cochrane Library were searched for relevant studies. Trials evaluating the effects of bergamot supplementation on cardiovascular outcomes in adults were included. Weighted mean differences (WMDs) were pooled using DerSimonian–Laird random‐effects models. The certainty of evidence was assessed using the Grading of Recommendations Assessment, Development and Evaluation (GRADE) approach. Nine RCTs involving 615 participants were included. Bergamot supplementation was significantly associated with reduced waist circumference (WMD = −0.96 cm; 95% CI: −1.91 to −0.01; *p* = 0.047), systolic blood pressure (WMD = −2.09 mmHg; 95% CI: −3.33 to −0.86; *p* = 0.001), glucose (WMD = −12.12 mg/dL; 95% CI: −17.05 to −7.19; *p* < 0.001), homeostatic model assessment of insulin resistance (WMD = −0.38 units; 95% CI: −0.65 to −0.11; *p* = 0.005), insulin (WMD = −2.93 IU/L; 95% CI: −5.06 to −0.79; *p* = 0.007), low‐density lipoprotein cholesterol (WMD = −39.53 mg/dL; 95% CI: −53.46 to −25.60; *p* < 0.001), total cholesterol (WMD = −44.69 mg/dL; 95% CI: −62.00 to −27.39; *p* < 0.001), and triglycerides (WMD = −52.47 mg/dL; 95% CI: −66.09 to −38.86; *p* < 0.001), while being associated with an increase in high‐density lipoprotein cholesterol (WMD = 5.63 mg/dL; 95% CI: 3.75 to 7.51; *p* < 0.001). Risk‐of‐bias assessment classified six trials as poor, two as fair, and one as good. According to the GRADE assessment, the certainty of evidence ranged from low to very low for all outcomes. Bergamot supplementation is associated with modest improvements in cardiometabolic risk factors, including lipid profile, glycemic control, systolic blood pressure, and waist circumference, in adults with dyslipidemia or metabolic syndrome. However, these observed effects are mainly based on very‐low‐certainty evidence, largely from studies at high risk of bias, and should not be interpreted as establishing the clinical efficacy of bergamot.

## Graphical abstract

Summary of the systematic review and meta‐analysis of randomized controlled trials evaluating the effects of bergamot ( *Citrus bergamia* ) supplementation on cardiometabolic risk factors in adults.

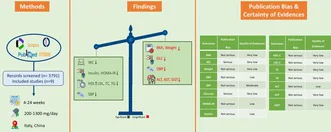

**Abbreviations**
ALT: Alanine Aminotransferase
AMPK: Adenosine Monophosphate‐activated Protein Kinase
AST: Aspartate Aminotransferase
BMI: Body Mass Index
CI: Confidence Interval
DBP: Diastolic Blood Pressure
FP: Fractional polynomial
GGT: Gamma‐Glutamyl Transferase
GRADE: Grading of Recommendations Assessment Development, and Evaluation
HDL‐C: High‐Density Lipoprotein Cholesterol
HOMA‐IR: Homeostatic Model Assessment of Insulin Resistance
LDL‐C: Low‐Density Lipoprotein Cholesterol
MeSH: Medical Subject Headings
PRISMA: Preferred Reporting Items for Systematic Reviews and Meta‐Analyzes
PROSPERO: International Prospective Register of Systematic Reviews
RCT: Randomized Controlled Trial
SBP: Systolic Blood Pressure
SDs: Standard Deviations
TC: Total Cholesterol
TG: Triglycerides
WC: Waist Circumference
WMD: Weighted Mean Difference

## 1 Introduction

Cardiovascular diseases remain the leading cause of global mortality, accounting for approximately 19.8 million deaths in 2022, with dyslipidemia, hypertension, and insulin resistance as key modifiable risk factors (WHO 2025). The growing prevalence of Type 2 diabetes mellitus, affecting over 537 million adults worldwide in 2021, further amplifies cardiovascular risk through mechanisms such as atherogenic dyslipidemia, endothelial dysfunction, and chronic inflammation (Magliano et al. 2021; Sun et al. 2022). These metabolic abnormalities including elevated low‐density lipoprotein cholesterol (LDL‐C) and triglycerides (TG), with reduced high‐density lipoprotein cholesterol (HDL‐C) highlight the need for more effective interventions (Einarson et al. 2018). While statins effectively reduce LDL‐C by up to 50% and decrease cardiovascular events, challenges such as statin intolerance, poor adherence, and persistent residual risk in high‐risk patients highlight the necessity for complementary non‐pharmacological strategies (Armitage et al. 2019; Banach et al. 2016).

Nutraceuticals, defined as food‐derived bioactive compounds, with therapeutic potential, have garnered significant interest for modulating cardiovascular risk factors as potential adjuncts to conventional risk‐reduction strategies (Cicero and Rizzo 2021). Bergamot ( *Citrus bergamia* ), a citrus fruit native to the Calabrian region of southern Italy, has been evaluated in human trials for its potential lipid‐lowering effects due to its high content of flavonoids and glycosides, such as neoeriocitrin, neohesperidin, naringin, and rutin (Nogata et al. 2006). These bioactive compounds have antioxidant, anti‐inflammatory, and lipid‐lowering properties in studies (Di Donna et al. 2009; Ferlazzo et al. 2016). However, human trials of bergamot supplementation have yielded inconsistent results regarding its effects on lipid profile, glycemic markers, and other cardiovascular risk factors, necessitating a comprehensive evidence synthesis to elucidate its efficacy and optimal dosing (Gliozzi et al. 2013; Mollace et al. 2011).

Previous systematic reviews and meta‐analyzes of bergamot supplementation have several limitations that preclude definitive clinical recommendations. Sadeghi‐dehsahraei et al. (2022) reported reductions in total cholesterol (TC), LDL‐C, and TG; however, their analysis included only five predominantly low‐quality studies, all conducted in Italy, and showed substantial heterogeneity without examining dose–response relationships (Sadeghi‐dehsahraei et al. 2022). Similarly, Lamiquiz‐Moneo et al. (2020) observed potential dose‐dependent effects, but only half of the included trials were randomized, and few adequately controlled for dietary changes, body weight, or other confounding factors (Lamiquiz‐Moneo et al. 2020). Variability in outcome reporting and statistical methods further limited interpretation (Sadeghi‐dehsahraei et al. 2022; Lamiquiz‐Moneo et al. 2020). Although Osadnik et al. identified bergamot as a potentially effective nutraceutical for reducing LDL‐C and TC, their estimates were based on only three trials and were supported by low‐certainty evidence according to Grading of Recommendations Assessment, Development and Evaluation (GRADE) (Osadnik et al. 2022). Moreover, previous reviews focused primarily on lipid outcomes and provided limited evidence regarding glycemic control and blood pressure.

Therefore, this systematic review and dose–response meta‐analysis aimed to evaluate the effects of bergamot supplementation on lipid profiles, glycemic markers, anthropometric measurements, liver‐function tests and blood pressure using evidence from randomized controlled trials. The review followed PRISMA 2020 guidelines and applied the GRADE framework to assess evidence certainty (Page et al. 2021). By examining dose–response relationships and a broader range of cardiometabolic outcomes, the study seeks to clarify bergamot's potential as a complementary intervention, including for high‐risk populations such as individuals with Type 2 diabetes.

## 2 Methods

### 2.1 Protocol and Registration

This systematic review and meta‐analysis was designed and reported in accordance with the Preferred Reporting Items for Systematic Reviews and Meta‐Analyzes 2020 (PRISMA 2020) statement (Page et al. 2021). The completed PRISMA checklist is provided in Supporting Information PRISMA Checklist, and the study‐selection process is presented in a PRISMA flow diagram. To promote transparency and accountability, the protocol was prospectively registered in the International Prospective Register of Systematic Reviews (PROSPERO) (CRD420251127505) before study initiation.

### 2.2 Search Strategy and Study Selection

A comprehensive literature search was conducted across major databases, including PubMed, Web of Science, Scopus, Embase, and the Cochrane Library, to identify relevant publications. The reference lists of eligible trials, relevant systematic reviews, and related meta‐analyzes were also manually screened to identify additional potentially eligible publications.

The search employed targeted terms such as “bergamot” combined with “randomized controlled trial,” drawn from controlled vocabularies like Medical Subject Headings (MeSH) terms in PubMed and Emtree in Embase. A customized search protocol was adapted for each database to optimize retrieval (detailed in Table S1). The search was restricted to publications in English, consistent with the eligibility criteria.

All identified records were imported into EndNote X7, and duplicate records were removed before screening. Two reviewers (M.R. and M.J.) independently screened titles and abstracts and subsequently assessed the full texts of potentially eligible reports. Reasons for excluding articles at the full‐text stage were documented and provided in Table S2. Disagreements were resolved through discussion and, when necessary, consultation with a third reviewer (H.M.).

### 2.3 Inclusion/Exclusion Criteria and Data Extraction

Studies were incorporated into this meta‐analysis if they satisfied predefined standards, including being original RCTs with either parallel or crossover configurations, featuring matched control arms, and being published in the English language. The research framework followed the PICO model: Population (individuals aged 18 years or older, irrespective of baseline health), Intervention (Bergamot and its polyphenolic extracts or formulations), Comparator (Placebo, dietary intervention/control, no intervention, or active comparator), and Outcomes (anthropometric measures, blood pressure, glycemic profile, lipid profile, and liver function tests).

Studies were required to provide sufficient quantitative data to calculate an effect estimate and its variance. Non‐randomized and quasi‐experimental studies, observational studies, animal or in vitro studies, reviews, editorials, commentaries, conference abstracts without sufficient data, and reports lacking usable pre‐intervention and post‐intervention outcome data were excluded. Studies published in languages other than English were also excluded.

Data were independently extracted by two reviewers (M.Ch. and M.R.) using a standardized and piloted extraction form. Extracted information included author, publication year, country, study design, participant characteristics, baseline health status, sample size, bergamot formulation, dose, intervention duration, comparator characteristics, outcome definitions, baseline and follow‐up means and standard deviations, change scores, and information required for risk‐of‐bias assessment. Disagreements were resolved through consensus or consultation with H.R.

When a trial contained more than one eligible bergamot intervention arm, the intervention arms with different doses were retained as separate comparisons rather than being combined. When multiple eligible intervention arms shared a common control group, the shared control sample size was divided equally across the relevant comparisons to avoid double‐counting of participants, while the original mean and standard deviation were retained.

### 2.4 Methodological Quality Assessment and Evidence Grading

The risk of bias in selected trials was appraised by two evaluators (M.R. and H.M.) employing the Cochrane Risk of Bias Tool (ROB1), with any disputes mediated by a third party (W.L.). The assessment included seven domains: (1) random sequence generation, (2) allocation concealment, (3) blinding of participants and personnel, (4) blinding of outcome assessment, (5) incomplete outcome data, (6) selective reporting, and (7) other sources of bias. Each domain was classified as having a low, high, or unclear risk of bias. The certainty of evidence for each outcome was independently assessed by V.U. and M.Ch. Disagreements were resolved through discussion or adjudication by A.J. using the GRADE approach and classified as high, moderate, low, or very low. The certainty of evidence was assessed across five GRADE domains: risk of bias, inconsistency, indirectness, imprecision, and publication bias. Risk of bias was rated as serious when more than 20% of the evidence for an outcome originated from trials judged to be at high risk of bias in at least one RoB 1 domain and these limitations were considered likely to influence the pooled estimate. Inconsistency was rated as serious when *I* ^2^ is equal or more than 50% and very serious when *I* ^2^ exceeded 75%, while also considering the direction and overlap of study‐specific confidence intervals. Indirectness was rated as serious when the geographical concentration limited the applicability of the evidence to the review question. Imprecision was rated as serious when the 95% confidence interval crossed the prespecified threshold of clinical importance or included both no effect and a potentially important effect. Publication bias was considered serious when Egger's tests indicated small‐study effects (*p* < 0.05).

### 2.5 Statistical Analysis

Analyzes were executed using Stata software, version 17.0 (StataCorp, College Station, TX). For each cardiovascular endpoint, mean values, standard deviations (SDs), and sample sizes were extracted from both bergamot and control groups at baseline and endpoint. The influence of bergamot was quantified via weighted mean differences (WMDs) in change scores, applying a random‐effects model with the DerSimonian‐Laird estimator to accommodate variability in study contexts. Because individual outcomes were reported using consistent measurement units, pooled effects were expressed as WMDs in change from baseline with corresponding 95% confidence intervals.

When change SDs were unavailable, they were imputed using the Follmann approach assuming a correlation coefficient (*R*) of 0.5. Standard errors were converted to SDs via SD = standard error × √n, with n as group size. Change SDs were derived as √ [(SD_baseline^2^ + SD_endpoint^2^) − (2 × *R* × SD_baseline × SD_endpoint)], where *R* represents the within‐participant correlation between baseline and follow‐up measurements. A correlation coefficient of 0.50 was used in the primary analysis.

Heterogeneity was quantified using the *I* ^2^ index and *τ* ^2^. *I* ^2^ values > 50% were considered substantial heterogeneity. To complement the relative measure of heterogeneity provided by *I* ^2^, the between‐study variance (*τ* ^2^) was reported for each outcome as an absolute measure of heterogeneity in the random‐effects framework (Veroniki et al. 2016). While *I* ^2^ expresses the proportion of total variability attributable to between‐study differences, *τ* ^2^ quantifies the actual variance of true effects across studies, and its square root (*τ*) represents the standard deviation of the distribution of true effects. Reporting *τ* ^2^ alongside *I* ^2^ is recommended because *I* ^2^ alone can be misleading, particularly when the number of studies is small or when confidence intervals are wide and *τ* ^2^ was estimated using the DerSimonian‐Laird method (Bakbergenuly et al. 2020). Sources of heterogeneity were probed through subgroup stratification. Subgroup analyzes were conducted based on the following comparisons: health status (metabolic syndrome vs. dyslipidemia/hyperlipidemia), country (Italy vs. China), intervention age (< 55 years vs. ≥ 55 years vs. not reported), baseline body mass index (BMI) (25–29.99 kg/m^2^ vs. ≥ 30 kg/m^2^ vs. not reported), duration of intervention (≤ 12 weeks vs. > 12 weeks), intervention type (tablet vs. capsule), formulations (bergamot vs. bergamot polyphenols), dose (< 800 mg/day vs. ≥ 800 mg/day), type of control (placebo vs. hypocaloric personalized diet vs. Rosuvastatin), sample size (< 50 vs. ≥ 50), and study quality (poor vs. fair vs. good).

Meta‐regression analyzes were performed to evaluate the effects of bergamot dosage and intervention duration on cardiovascular risk parameters. A non‐linear dose–response approach using a fractional polynomial (FP) random‐effects model was utilized to investigate the association between different supplementation doses, durations and health outcomes, to characterize potential dose‐ and duration‐response relationships. FP models were selected because they provide a flexible approach for modeling potential non‐linear relationships while accommodating studies with a limited number of exposure levels (Orsini et al. 2006; Xu and Doi 2018). To assess the stability of the results, sensitivity analyzes, including iterative exclusion of individual studies, were performed to determine the impact of each study on the pooled effect size. Potential publication bias was explored through the application of Egger's statistical test (Egger et al. 1997). To check for missing‐study bias, we ran a trim‐and‐fill analysis on each outcome. This method looks at the funnel plot, trims off any studies causing asymmetry, then estimates what the pooled effect would look like if those “missing” studies were added back in. It's a useful way to gauge whether publication bias might be skewing the results, and to see how much the overall effect size shifts once that's accounted for (Shi and Lin 2019).

## 3 Results

### 3.1 Study Selection

**FIGURE 1:**
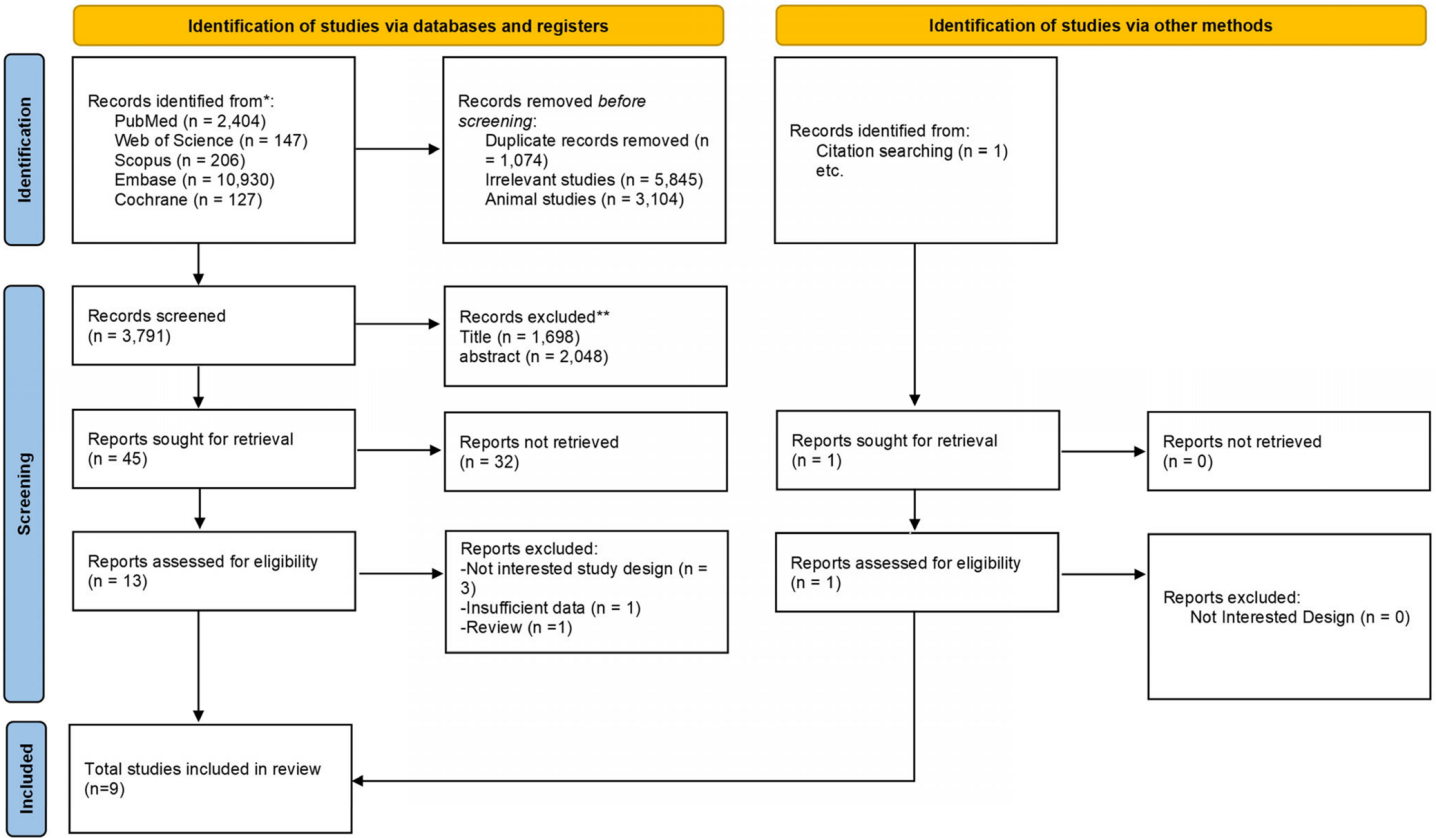
Flowchart of study selection for inclusion trials in the systematic review.

The selection process of the included studies is presented in Figure 1. A database search resulted in identifying a total of 13,814 studies, including PubMed (*n* = 2404), ISI Web of Science (*n* = 147), Scopus (*n* = 206), Embase (*n* = 10,930) and Cochrane Library (*n* = 127). A total of 1074 duplicate, 5845 irrelevant, and 3104 animal studies were excluded, and 3791 studies were screened based on title and abstract. After screening, 3746 irrelevant studies were excluded, and 45 full‐text studies were considered. Of these, 32 were not retrievable and 5 were excluded with a reason, and one study was added by manual search. Table S2 shows the studies that were excluded from the full‐text review and the reasons for exclusion. As a result, 9 studies with 615 participants met the inclusion criteria (Gliozzi et al. 2013; Mollace et al. 2011; Cai et al. 2017; Capomolla et al. 2019; Di Folco et al. 2018; Fogacci et al. 2023; Mollace et al. 2019; Rondanelli et al. 2021; Spina et al. 2024).

### 3.2 Study Characteristics

**FIGURE 2:**
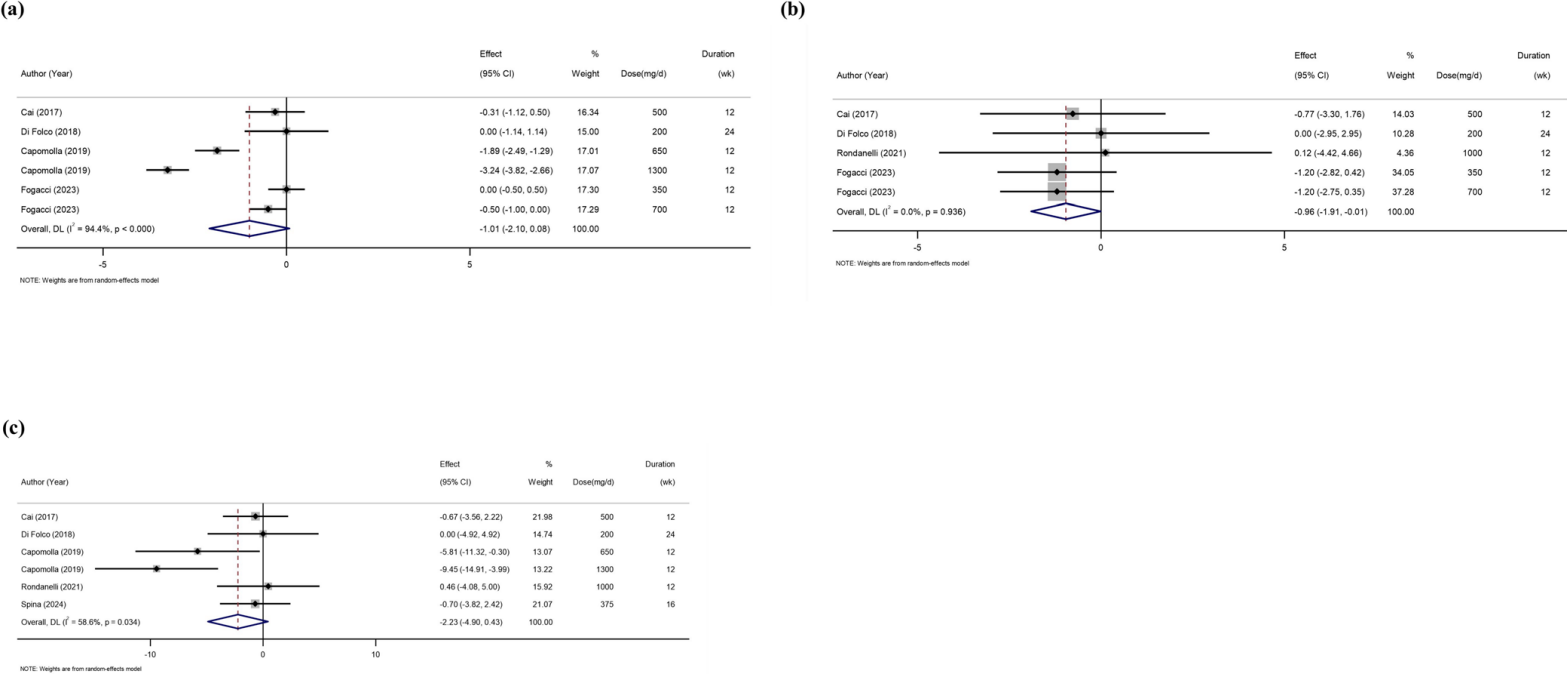
Forest plot of the effects of Bergamot supplement on anthropometric measures (a: Body mass index, b: Waist circumference, c: Weight).

**FIGURE 3:**
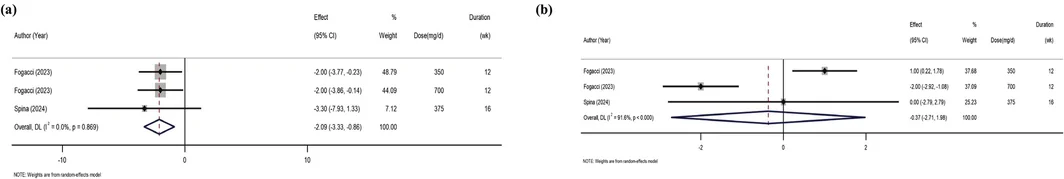
Forest plot of the effects of Bergamot supplement on blood pressure (a: Systolic blood pressure, b: Diastolic blood pressure).

**FIGURE 4:**
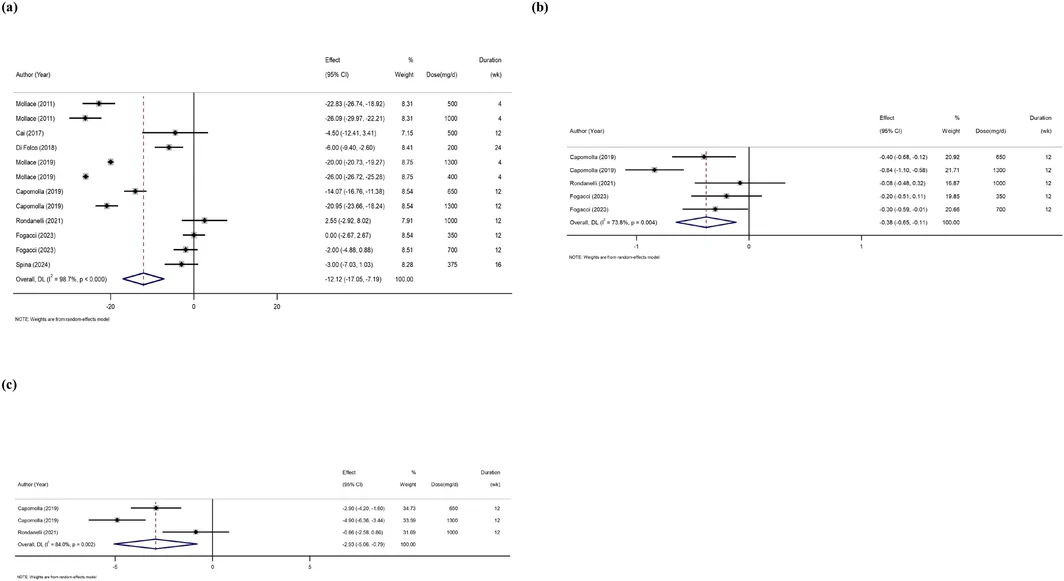
Forest plot of the effects of Bergamot supplement on glycemic profile (a: Glucose, b: Homeostatic model assessment of insulin resistance, c: Insulin).

**FIGURE 5:**
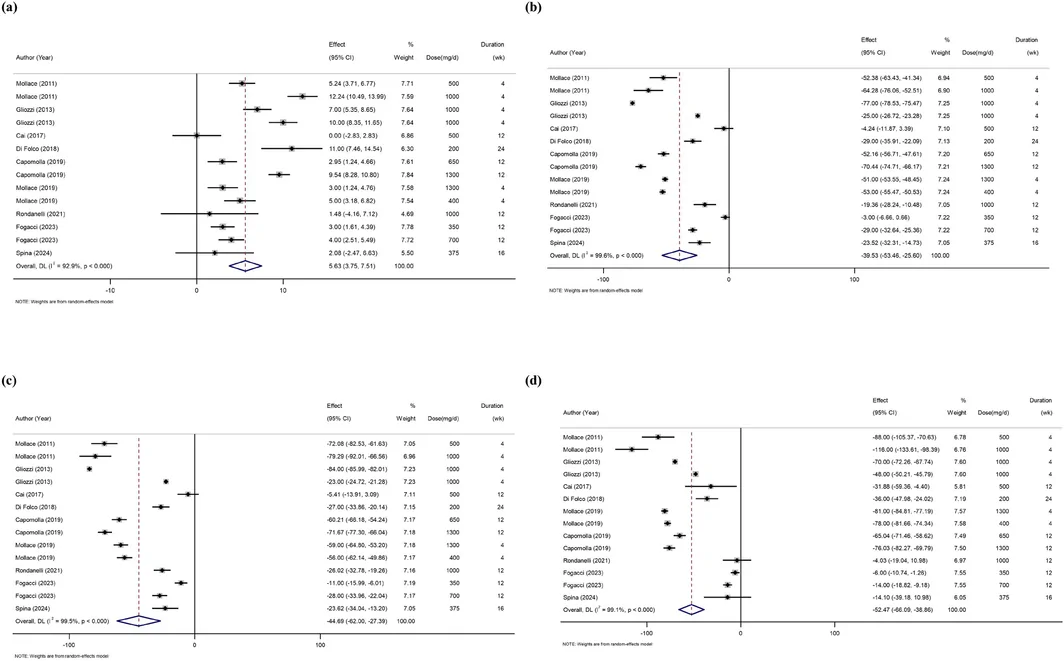
Forest plot of the effects of Bergamot supplement on lipid profile (a: High‐density lipoprotein cholesterol, b: Low‐density lipoprotein cholesterol, c: Total cholesterol, d: Triglycerides).

**FIGURE 6:**
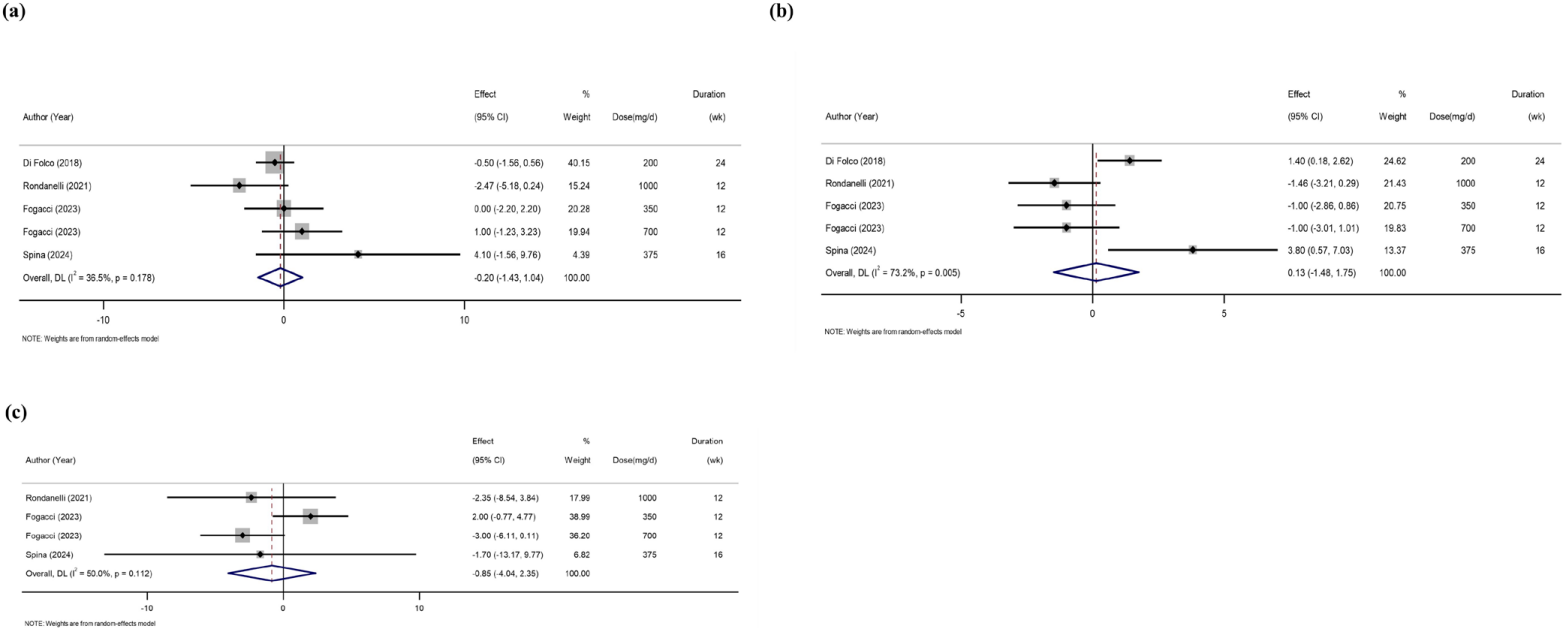
Forest plot of the effects of Bergamot supplement on liver function tests (a: Alanine aminotransferase, b: Aspartate aminotransferase, c: Gamma‐glutamyl transferase).

The characteristics of included studies are presented in Table 1. The WMD and 95% confidence interval (CI) of BMI, weight, waist circumference (WC), diastolic blood pressure (DBP), systolic blood pressure (SBP), glucose, homeostatic model assessment of insulin resistance (HOMA‐IR), insulin, HDL‐C, LDL‐C, TC, TG, alanine aminotransferase (ALT), aspartate aminotransferase (AST), gamma‐glutamyl transferase (GGT) and their changes in comparison with control groups are presented in Figures 2, 3, 4, 5, 6 respectively. The nine included RCTs were published between 2011 and 2024 and were carried out in Italy (Gliozzi et al. 2013; Mollace et al. 2011; Capomolla et al. 2019; Di Folco et al. 2018; Fogacci et al. 2023; Mollace et al. 2019; Rondanelli et al. 2021; Spina et al. 2024) and China (Cai et al. 2017). In the intervention and control group, the participant ages ranged from 20 to 75 years old. Bergamot formulations and dosages varied, ranging from 200 mg/d to 1300 mg/d. The duration of intervention was between 4 and 24 weeks. Sample sizes in the intervention group ranged from 15 to 48 participants. All studies included both sexes. Studies included participants with dyslipidemia (Gliozzi et al. 2013; Mollace et al. 2011; Cai et al. 2017; Mollace et al. 2019; Rondanelli et al. 2021; Spina et al. 2024) and participants with metabolic syndrome (Capomolla et al. 2019; Di Folco et al. 2018; Fogacci et al. 2023).

**TABLE 1:** Characteristics of included studies in the meta‐analysis.

Studies. year (Ref.)	Country	Study design	Participant	Sample size (Sex)	Sample size INT/CON	Trial duration (Week)	Means Age INT/CON	Dose of supplement (mg/d)	Type of supplement INT/CON	Outcomes
Mollace et al. (2011)	Italy	RCT, DB	Hyperlipemia ± hyperglycaemia	59 (B)	20/20 19/20	4	—	500 1000	Bergamot/Placebo	Glucose, HDL‐C, LDL‐C, TC, TG
Gliozzi et al. (2013)	Italy	RCT	Elevated LDL‐C & TG	60 (B)	15/15 15/15	4	—	1000	BPF + Rosuvastatin/Rosuvastatin Or BPF/Placebo	HDL‐C, LDL‐C, TC, TG
Cai et al. (2017)	China	RCT, DB	Dyslipidemia	98 (B)	48/50	12	66.7 ± 9.4/63.8 ± 8.6	500	*Citrus bergamia* /Placebo (soy oil)	Weight, BMI, WC, Glucose, HDL‐C, LDL‐C, TC, TG
Di Folco et al. (2018)	Italy	RCT	Overweight adults with metabolic syndrome	80 (B)	40/40	24	45 ± 5/44 ± 3	200	Bergamot + diet/Hypocaloric diet	Weight, BMI, WC, Glucose, HDL‐C, LDL‐C, TC, TG, ALT, AST
Capomolla et al. (2019)	Italy	RCT, DB	Metabolic syndrome	44 (B)	15/14 15/14	12	56.1 ± 10.9/55.6 ± 7.4, 59 ± 7.6/55.6 ± 7.4	650 1300	Bergamot polyphenols/Placebo (maltodextrin)	Weight, BMI, Glucose, GGT, HOMA‐IR, Insulin, HDL‐C, LDL‐C, TC, TG
Mollace et al. (2019)	Italy	RCT, DB	Mixed hyperlipidemia + T2DM	60 (B)	20/20 20/20	4	50 ± 12/52 ± 11, 51 ± 12/52 ± 11	400, 1300	BPF/Placebo/BPF phytosome/Placebo	Glucose, HDL‐C, LDL‐C, TC, TG
Rondanelli et al. (2021)	Italy	RCT, DB	Overweight and obese Class I subject with mild hypercholesterolemia	64 (B)	33/31	12	59.03/57.29	1000	Bergamot powder/Placebo	Weight, BMI, WC, Glucose, Insulin, HOMA‐IR, GGT, HDL‐C, LDL‐C, TC, TG, ALT, AST
Fogacci et al. (2023)	Italy	RCT, DB	Metabolic syndrome	90 (B)	30/30 30/30	12	20–75/20–75	350 700	Bergamot/Placebo	BMI, WC, Glucose, HOMA‐IR, GGT, HDL‐C, LDL, TC, TG, ALT, AST, SBP, DBP
Spina et al. (2024)	Italy	RCT, DB	Healthy with untreated dyslipidemia	60 (B)	30/30	16	50.9/51.5	375	Bergavit/Placebo	Weight, Glucose, GGT, HDL‐C, LDL, TC, TG, ALT, AST, SBP, DBP

Abbreviations: ALT, Alanine Aminotransferase; AST, Aspartate Aminotransferase; B, Both Sex; BMI, Body Mass Index; BPF, Bergamot Polyphenolic Fraction; CON, Control Group; DB, Double‐Blinded; GGT, Gamma‐Glutamyl Transferase; HDL‐C, High‐Density Lipoprotein Cholesterol; HOMA‐IR, Homeostatic Model Assessment of Insulin Resistance; INT, Intervention Group; LDL‐C, Low‐Density Lipoprotein Cholesterol; RCT, Randomized Controlled Trial; T2DM, Type 2 Diabetes Mellitus; TB, Triple‐Blinded; TC, Total Cholesterol; TG, Triglycerides; WC, Waist Circumference.

The sample sizes for the intervention and control groups across different outcomes are as follows: BMI: *n* = 312 (intervention: 178, control: 134), WC: *n* = 332 (intervention: 181, control: 151), weight: *n* = 346 (intervention: 181, control: 165), DBP: *n* = 150 (intervention: 90, control: 60), SBP: *n* = 150 (intervention: 90, control: 60), glucose: *n* = 555 (intervention: 320, control: 235), HOMA‐IR: *n* = 198 (intervention: 123, control: 75), insulin: *n* = 108 (intervention: 63, control: 45), HDL‐C: *n* = 615 (intervention: 350, control: 265), LDL‐C: *n* = 615 (intervention: 350, control: 265), TC: *n* = 615 (intervention: 350, control: 265), TG: *n* = 615 (intervention: 350, control: 265), ALT: *n* = 294 (intervention: 163, control: 131), AST: *n* = 294 (intervention: 163, control: 131), and GGT: *n* = 214 (intervention: 123, control: 91).

### 3.3 Qualitative Data Assessment

Based on the Cochrane Risk of Bias Assessment tool, six studies were classified as poor quality (Gliozzi et al. 2013; Cai et al. 2017; Capomolla et al. 2019; Di Folco et al. 2018; Fogacci et al. 2023; Mollace et al. 2019), two studies as fair quality (Mollace et al. 2011; Spina et al. 2024) and one study as good quality (Rondanelli et al. 2021) (Table 2).

**TABLE 2:** Quality of included studies in the meta‐analysis.

Study, year (Ref.)	Random sequence generation	Allocation concealment	Blinding of participants & personnel	Blinding of outcome assessment	Incomplete outcome data	Selective outcome reporting	Other sources of bias	Overall quality
Mollace et al. (2011)	L	H	L	U	U	L	L	Fair
Gliozzi et al. (2013)	U	H	H	H	L	L	L	Poor
Cai et al. (2017)	L	H	L	H	L	L	L	Poor
Di Folco et al. (2018)	U	H	H	H	L	L	L	Poor
Capomolla et al. (2019)	U	H	U	H	H	L	L	Poor
Mollace et al. (2019)	U	H	U	H	U	L	L	Poor
Rondanelli et al. (2021)	L	L	L	L	L	L	L	Good
Fogacci et al. (2023)	L	U	U	U	L	L	L	Poor
Spina et al. (2024)	L	U	L	U	L	L	L	Fair

Abbreviations: H, high risk of bias; L, low risk of bias; U, unclear risk of bias.

### 3.4 Effects of Bergamot Supplementation on Anthropometric Measures

Figure 2 delineates the impact of bergamot supplementation on anthropometric measures in comparison with control groups. Out of four studies with six arms on the effect of bergamot supplementation on BMI in comparison with control groups, no significant effect was observed (WMD = −1.01 kg/m^2^, 95% CI: −2.10, 0.08; *p* = 0.069). Moreover, the heterogeneity among studies was significant (*I* ^2^: 94.4%, *p* < 0.001). The between‐study variance (*τ* ^2^) for BMI was 1.729 (*τ* = 1.32 kg/m^2^), indicating substantial variability in true effects across studies.

In the case of four trials (five arms) exploring the impact of bergamot supplementation on WC, the meta‐analysis indicated a small reduction in WC compared to the control group (WMD = −0.96 cm; 95% CI: −1.91, −0.01; *p* = 0.047) and there was no observed heterogeneity among these studies (*I* ^2^: 0.0%, *p* = 0.936). For WC, *τ* ^2^ = 0.000, indicating that studies estimated a consistent true effect, with prediction intervals approximating the confidence intervals.

Five trials (six arms) were dedicated to examining the effect of bergamot supplementation on weight. The meta‐analysis showed no significant effect on weight (WMD = −2.23 kg; 95% CI: −4.90, 0.43; *p* = 0.101), and a moderate heterogeneity was observed among these studies (*I* ^2^: 58.6%, *p* = 0.034). For body weight, *τ* ^2^ = 6.235 (*τ* = 2.50 kg), reflecting substantial between‐study variance and wide prediction intervals, limiting the interpretability of the pooled estimates.

Sensitivity analysis revealed that excluding one arm of the Fogacci et al. study (2023), in which patients received 350 mg/day of bergamot, significantly changed the overall effect on BMI (WMD: −1.22 kg/m^2^; 95% CI: −2.44, −0.004). For WC, the results significantly changed after excluding both arms of Fogacci et al. (2023) (WMD = −0.83 cm; 95% CI: −2.00, 0.33 and WMD = −0.82 cm; 95% CI: −2.01, 0.38) and Cai et al. (2017) (WMD = −0.99 cm; 95% CI: −2.01, 0.03). However, excluding any study did not affect the overall results for body weight.

Additionally, there was no evidence of publication bias for BMI (Egger's *p* = 0.929) or weight (Egger's *p* = 0.205), whereas Egger's test suggested potential small‐study effects for WC (Egger's *p* = 0.022). Trim‐and‐fill analysis showed no evidence of publication bias for either weight or BMI, as no studies were imputed for Weight (*g* = −0.239, 95% CI: −0.561 to 0.082) or BMI (*g* = −0.340, 95% CI: −0.575 to −0.105); WC showed minor potential bias, with one imputed study slightly attenuating the effect from *g* = −0.150 to g = −0.102 (95% CI: −0.306 to 0.103).

### 3.5 Effects of Bergamot Supplementation on Blood Pressure

Meta‐analysis of two studies providing three arms revealed a significant reduction in SBP following bergamot supplementation with low heterogeneity among included studies (WMD = −2.09 mmHg; 95% CI: −3.33 to −0.86; *p* = 0.001) (*I* ^2^: 0.0%, *p* = 0.869). In contrast, no significant change was observed for DBP (WMD = −0.37 mmHg; 95% CI: −2.71 to 1.98; *p* = 0.760), and heterogeneity among studies was significant (*I* ^2^: 91.6%, *p* < 0.001). For SBP, *τ* ^2^ = 0.000, indicating that studies estimated a consistent true effect, with prediction intervals approximating the confidence intervals and for DBP, *τ* ^2^ = 3.634 (*τ* = 1.91 mmHg), reflects substantial between‐study variance.

Excluding any individual study did not alter the overall results for SBP. However, excluding the 700 mg/day bergamot treatment arm from Fogacci et al. study (2023) significantly affected DBP, resulting in a notable change (WMD = 0.93 mmHg; 95% CI: 0.17 to 1.68). Details are presented in Figure 3. Moreover, no indication of publication bias was detected for DBP (Egger's *p* = 0.92); however, evidence of potential publication bias was observed for SBP (Egger's *p* = 0.039). Although Egger's test suggested potential asymmetry for SBP, trim‐and‐fill analysis identified no missing studies (*g* = −0.546, 95% CI: −0.878 to −0.214). This discrepancy likely reflects the limited statistical power of both tests given the small number of included studies (*k* = 3), and results should therefore be interpreted with caution rather than as definitive evidence of publication bias.

### 3.6 Effects of Bergamot Supplementation on Glycemic Profile

Figure 4 presents an overview of the impact of bergamot supplementation on glycemic‐related parameters. In total, 8 studies (12 arms) assessed glucose, and meta‐analysis revealed a significant effect of bergamot supplementation on glucose compared with control groups (WMD = −12.12 mg/dL; 95% CI: −17.05, −7.19; *p* < 0.001), with high heterogeneity observed (*I* ^2^: 98.7%, *p* < 0.001). The between‐study variance (*τ* ^2^) for glucose was 72.158 (*τ* = 8.49 mg/dL), indicating very large variability in true effects across studies.

Three RCTs with 5 arms evaluated HOMA‐IR, revealing a significant decrease with bergamot supplementation (WMD = −0.38 units; 95% CI: −0.65, −0.11; *p* = 0.005), with moderate heterogeneity among the included studies (*I* ^2^: 73.8%, *p* = 0.004). The between‐study variance (*τ* ^2^) for HOMA‐IR was 0.067 (*τ* = 0.259 units), indicating modest between‐study variability that is small relative to the pooled effect, supporting a reasonably consistent direction of effect across studies.

Two trials with three arms examined insulin, and pooled analysis indicated a significant reduction with bergamot supplementation compared with control groups (WMD = −2.93 IU/L; 95% CI: −5.06, −0.79; *p* = 0.007), with considerable heterogeneity among the studies (*I* ^2^: 84.0%, *p* = 0.002). The between‐study variance (*τ* ^2^) for insulin was 2.989 (*τ* = 1.729 IU/L), indicating substantial between‐study variability.

Upon exclusion of any individual study, the overall effects of bergamot on glucose and HOMA‐IR remained unchanged. However, removing two arms of Capomolla et al. (2019) significantly changed the results of bergamot supplementation on insulin levels, yielding WMDs of −2.90 IU/L (95% CI: −6.86, 1.05) for one arm and −1.96 IU/L (95% CI: −3.95, 0.03) for the other arm.

Egger's test suggested potential small‐study effects for glucose (*p* = 0.017), whereas no evidence of small‐study effects was observed for HOMA‐IR (*p* = 0.125) or insulin (*p* = 0.665). Trim‐and‐fill detected no missing studies for FBS (*g* = −5.432, 95% CI: −9.957 to −0.907), Insulin (*g* = −1.762, 95% CI: −3.419 to −0.106), or HOMA‐IR (*g* = −0.951, 95% CI: −1.854 to −0.048).

### 3.7 Effects of Bergamot Supplementation on Lipid Profile

Figure 5 provides an overview of the impact of bergamot supplements on the lipid profile. The combined effect size of nine studies with 14 arms revealed a significant increase in HDL‐C concentration with the use of bergamot supplements compared to control groups (WMD = 5.63 mg/dL; 95% CI: 3.75, 7.51; *p* < 0.001), with high heterogeneity observed among the studies (*I* ^2^: 92.9%, *p* < 0.001). The between‐study variance (*τ* ^2^) for HDL‐C was 11.298 (*τ* = 3.36 mg/dL), indicating substantial between‐study variability.

A total of nine studies with 14 arms and 615 participants investigated the impact of bergamot supplements on LDL‐C levels. The meta‐analysis results demonstrated a noteworthy reduction in LDL‐C in the intervention group compared to the control group (WMD = −39.53 mg/dL; 95% CI: −53.46, −25.60; *p* < 0.001), although high heterogeneity was observed among the studies (*I* ^2^: 99.6%, *p* < 0.001). The between‐study variance (*τ* ^2^) for LDL‐C was 696.34 (*τ* = 26.39 mg/dL), reflecting extreme variability across studies.

The effect of bergamot supplements on TC levels in comparison with the control group was assessed in nine trials including 14 arms. The WMD analysis indicated a significant effect on TC (WMD = −44.69 mg/dL; 95% CI: −62.00, −27.39; *p* < 0.001), with observed heterogeneity among the studies (*I* ^2^: 99.5%, *p* < 0.001). The between‐study variance (*τ* ^2^) for TC was 1100 (*τ* = 33.17 mg/dL), indicating extreme between‐study variability.

Nine trials (14 arms) reported the effect of bergamot supplements on TG concentration. The meta‐analysis highlighted a significant reduction in TG levels due to bergamot supplements in comparison with the control group (WMD = −52.47 mg/dL; 95% CI: −66.09, −38.86; *p* < 0.001), and high heterogeneity was observed among the studies (*I* ^2^: 99.1%, *p* < 0.001). The between‐study variance (*τ* ^2^) for TG was 633.617 (*τ* = 25.17 mg/dL), reflecting very large between‐study variability.

Sensitivity analyzes for HDL‐C, LDL‐C, TC and TG revealed that excluding any single study did not significantly alter the pooled effect size.

Moreover, there was no identified evidence of publication bias for HDL‐C (Egger's *p* = 0.530), LDL‐C (Egger's *p* = 0.302), TC (Egger's *p* = 0.850) or TG (Egger's *p* = 0.664). Regarding trim‐and‐fill analysis, no imputation was needed for TG (*g* = −6.896), LDL (*g* = −8.058), HDL (*g* = 2.018), or TC (*g* = −6.107), all with no evidence of publication bias.

### 3.8 Effects of Bergamot Supplementation on Liver Function Tests

The effects of bergamot supplementation on ALT and AST were assessed in four RCTs including 294 patients and 5 arms as presented in Figure 6. According to the meta‐analysis, bergamot supplementation did not exhibit a significant effect on ALT compared with control (WMD = −0.20 IU/L; 95% CI: −1.43, 1.04; *p* = 0.754), or AST (WMD = 0.13 IU/L; 95% CI: −1.48, 1.75; *p* = 0.871). Heterogeneity was low for ALT (*I* ^2^: 36.5%, *p* = 0.178) and moderate for AST (*I* ^2^: 73.2%, *p* = 0.005). The between‐study variance (*τ* ^2^) for ALT and AST was 0.700, 2.381 (*τ* = 0.837, 1.543 IU/L), indicating modest between‐study variability. The effect of bergamot supplementation on GGT compared to the control group was assessed in three RCTs including 214 patients and 4 arms. According to the analysis, bergamot supplementation did not exhibit a significant effect on GGT in comparison with the control group (WMD = −0.85 IU/L; 95% CI: −4.04, 2.35; *p* = 0.605). Notably, moderate heterogeneity was observed among the included studies (*I* ^2^: 50.0%, *p* = 0.112). The between‐study variance (*τ* ^2^) for GGT was 4.842 (*τ* = 2.200 IU/L), indicating moderate between‐study variability.

Sensitivity analysis indicated that removing the 350 mg/day treatment arm from Fogacci et al. (2023), significantly changed the results for GGT (WMD = −2.80 IU/L; 95% CI: −5.51, −0.10).

In addition, there was no identified evidence of publication bias for ALT (Egger's *p* = 0.512), AST (Egger's *p* = 0.960) and GGT (*p* = 0.686). Based on the trim‐and‐fill analysis, AST (*g* = 0.035) and ALT (*g* = −0.021) both showed no missing studies; however, given the small number of contributing trials, these findings should be interpreted cautiously.

### 3.9 Subgroup Analysis

Subgroup analyzes were performed to find sources of heterogeneity and to investigate whether the pooled effects of bergamot supplementation varied according to clinical and methodological characteristics (Table 3). Participants were categorized by health conditions, country, age, BMI, duration of intervention, intervention type, formulations, dosage, control type, sample size, and study quality. Health conditions were classified as metabolic syndrome or dyslipidemia/hyperlipidemia. Studies were also grouped according to whether they were conducted in Italy or China. Mean participant age was categorized as < 55 years, ≥ 55 years, or not reported (NR), whereas BMI was classified as overweight (25 ≤ BMI < 30 kg/m^2^), obese (BMI ≥ 30 kg/m^2^), or NR. Intervention duration was divided into ≤ 12 weeks and > 12 weeks. Additional subgroup categories included tablets versus capsules, general bergamot preparations versus bergamot polyphenolic fraction (BPF), doses < 800 mg/day versus ≥ 800 mg/day, and placebo versus hypocaloric personalized diet or rosuvastatin comparators. Studies were further classified according to sample size (< 50 or ≥ 50 participants) and methodological quality (poor, fair, or good).

**TABLE 3:** Description of the analysis and subgroup results of Bergamot supplementation on cardiovascular disease risk factors.

	Study arms *N*	Participant *N*	WMD (95% CI)	*p*	Heterogeneity			
					*p* heterogeneity	*I* ^2^	*p* between sub‐groups	Between‐study variance (τ^*2*^)
**Analysis and subgroup results of Bergamot supplementation on BMI**								
Overall effect	6	312	−1.01 (−2.10, 0.08)	0.069	< 0.001	94.4%		1.729
Health status								
Metabolic syndrome	5	214	−1.15 (−2.40, 0.11)	0.074	< 0.001	95.4%	0.272	
Dyslipidemia/Hyperlipidemia	1	98	−0.31 (−1.12, 0.50)	0.452	—	—		
Country								
Italy	5	214	−1.15 (−2.40, 0.11)	0.074	0.00	95.4%	0.272	
China	1	98	−0.31 (−1.12, 0.50)	0.452	—	—		
Intervention age (years)								
< 55	3	170	−0.23 (−0.57, 0.12)	0.204	0.354	3.8%	0.049	
≥ 55	3	142	−1.83 (−3.40, −0.27)	**0.021**	< 0.001	94.1%		
Baseline BMI (kg/m^2^)								
25 ≤ BMI < 30	4	210	−0.67 (−1.50, 0.15)	0.108	< 0.001	87.4%	0.556	
BMI ≥ 30	2	102	−1.66 (−4.83, 1.52)	0.306	< 0.001	95.9%		
Duration (weeks)								
≤ 12	5	232	−1.19 (−2.40, 0.02)	0.053	< 0.001	95.4%	0.161	
> 12	1	80	0.00 (−1.14, 1.14)	1.000	—	—		
Intervention type								
Tablet	3	170	−0.23 (−0.57, 0.12)	0.204	0.354	3.8%	0.049	
Capsule	3	142	−1.83 (−3.40, −0.27)	**0.021**	< 0.001	94.1%		
Formulations								
Bergamot	4	268	−0.24 (−0.55, 0.07)	0.134	0.549	0.0%	0.001	
Bergamot Polyphenols	2	44	−2.57 (−3.89, −1.24)	**< 0.001**	0.002	90%		
Dose (mg/day)								
< 800	5	290	−0.57 (−1.30, 0.16)	0.124	< 0.001	83.9%	< 0.001	
≥ 800	1	22	−3.24 (−3.82, −2.66)	**< 0.001**	—	—		
Control type								
Placebo	5	232	−1.19 (−2.40, 0.02)	0.053	< 0.001	95.4%	0.161	
Hypocaloric personalized diet	1	80	0.00 (−1.14, 1.14)	1.000	—	—		
Sample size								
< 50	4	178	−1.40 (−2.82, 0.02)	0.053	< 0.001	96.3%	0.135	
≥ 50	2	134	−0.21 (−0.87, 0.45)	0.539	0.664	0.0%		
**Analysis and subgroup results of Bergamot supplementation on WC**								
Overall effect	5	332	−0.96 (−1.91, −0.01)	**0.047**	0.936	0.0%		0.0
Health status								
Metabolic Syndrome	3	170	−1.05 (−2.10, −0.001)	**0.050**	0.758	0.0%	0.695	
Dyslipidemia/Hyperlipidemia	2	162	−0.56 (−2.77, 1.65)	0.620	0.737	0.0%		
Country								
Italy	4	234	−0.99 (−2.01, 0.03)	0.058	0.850	0.0%	0.875	
China	1	98	−0.77 (−3.30, 1.76)	0.551	—	—		
Intervention age (years)								
< 55	3	170	−1.05 (−2.10, −0.00)	**0.050**	0.758	0.0%	0.695	
≥ 55	2	162	−0.56 (−2.77, 1.65)	0.620	0.737	0.0%		
Baseline BMI (kg/m^2^)								
25 ≤ BMI < 30	4	252	−1.07 (−2.07, −0.07)	**0.036**	0.946	0.0%	0.502	
BMI ≥ 30	1	80	0.00 (−2.95, 2.95)	1.000	—	—		
Duration (weeks)								
≤ 12	4	252	−1.07 (−2.07, −0.07)	**0.036**	0.946	0.0%	0.502	
> 12	1	80	0.00 (−2.95, 2.95)	1.000	—	—		
Intervention type								
Tablet	4	234	−0.99 (−2.01, 0.03)	0.058	0.850	0.00%	0.875	
Capsule	1	98	−0.77 (−3.30, 1.76)	0.551	—	—		
Dose (mg/day)								
< 800	4	252	−1.01 (−1.98, −0.04)	**0.041**	0.898	0.0%	0.634	
≥ 800	1	80	0.12 (−4.42, 4.66)	0.959	—	—		
Control type								
Placebo	4	252	−1.07 (−2.07, −0.07)	**0.036**	0.946	0.0%	0.502	
Hypocaloric personalized diet	1	80	0.00 (−2.95, 2.95)	1.000	—	—		
Sample size								
< 50	2	90	−1.20 (−2.32, −0.08)	**0.036**	1.000	—	0.431	
≥ 50	3	242	−0.36 (−2.13, 1.41)	0.691	0.904	0.0%		
Quality of study								
Poor	4	252	−1.01 (−1.98, −0.04)	**0.041**	0.898	0.00%	0.634	
Good	1	80	0.12 (−4.42, 4.66)	0.959	—	—		
**Analysis and subgroup results of Bergamot supplementation on Weight**								
Overall effect	6	346	−2.23 (−4.90, 0.43)	0.101	0.034	58.6%		6.235
Health status								
Metabolic Syndrome	3	124	−4.98 (−10.52, 0.56)	0.078	0.037	69.5%	0.132	
Dyslipidemia/Hyperlipidemia	3	222	−0.48 (−2.40, 1.44)	0.625	0.905	0.0%		
Country								
Italy	5	248	−2.77 (−6.24, 0.70)	0.117	0.022	64.9%	0.361	
China	1	98	−0.67 (−3.56, 2.22)	0.650	—	—		
Intervention age (years)								
< 55	2	140	−0.50 (−3.14, 2.14)	0.711	0.814	0.0%	0.241	
≥ 55	4	206	−3.48 (−7.71, 0.75)	0.107	0.013	72.2%		
Baseline BMI (kg/m^2^)								
25 ≤ BMI < 30	4	244	−1.10 (−3.07, 0.87)	0.272	0.334	11.8%	0.463	
BMI ≥ 30	2	102	−4.65 (−13.91, 4.61)	0.325	0.012	84.3%		
Duration (weeks)								
≤ 12	4	206	−3.48 (−7.71, 0.75)	0.107	0.013	72.2%	0.241	
> 12	2	140	−0.50 (−3.14, 2.14)	0.711	0.814	0.0%		
Intervention type								
Tablet	2	158	0.25 (−3.09, 3.59)	0.884	0.893	0.0%	0.139	
Capsule	4	188	−3.53 (−7.25, 0.20)	0.064	0.016	71.1%		
Formulations								
Bergamot	4	302	−0.42 (−2.21, 1.38)	0.649	0.972	0.0%	0.001	
Bergamot Polyphenols	2	44	−7.65 (−11.53, −3.77)	**< 0.001**	0.358	0.0%		
Dose (mg/day)								
< 800	4	260	−1.18 (−3.07, 0.72)	0.223	0.371	4.4%	0.526	
≥ 800	2	86	−4.38 (−14.08, 5.33)	0.377	0.006	86.6%		
Control type								
Placebo	5	266	−2.69 (−5.80, 0.42)	0.090	0.021	65.5%	0.365	
Hypocaloric personalized diet	1	80	0.00 (−4.92, 4.92)	1.000	—	—		
Sample size								
< 50	2	44	−7.65 (−11.53, −3.77)	**< 0.001**	0.358	0.0%	0.001	
≥ 50	4	302	−0.42 (−2.21, 1.38)	0.649	0.972	0.0%		
Quality of study								
Poor	4	222	−3.63 (−7.83, 0.57)	0.090	0.01	70.4%	0.387	
Fair	1	60	−0.70 (−3.83, 2.43)	0.661	—	—		
Good	1	64	0.46 (−4.09, 5.10)	0.843	—	—		
**Analysis and subgroup results of Bergamot supplementation on DBP**								
Overall effect	3	150	−0.37 (−2.71, 1.98)	0.760	< 0.001	91.6%		3.634
Health status								
Metabolic Syndrome	2	90	−0.49 (−3.43, 2.45)	0.744	< 0.001	95.8%	0.81	
Dyslipidemia/Hyperlipidemia	1	60	0.00 (−2.79, 2.79)	1.000	—	—		
Duration (weeks)								
≤ 12	2	90	−0.49 (−3.43, 2.45)	0.744	< 0.001	95.8%	0.813	
> 12	1	60	0.00 (−2.79, 2.79)	1.000	—	—		
Intervention type								
Tablet	2	90	−0.49 (−3.43, 2.45)	0.744	< 0.001	95.8%	0.813	
Capsule	1	60	0.00 (−2.79, 2.79)	1.000	—	—		
Sample size								
< 50	2	90	−0.49 (−3.43, 2.45)	0.744	< 0.001	95.8%	0.813	
≥ 50	1	60	0.00 (−2.79, 2.79)	1.000	—	—		
Quality of study								
Poor	2	90	−0.49 (−3.43, 2.45)	0.744	< 0.001	95.8%	0.813	
Fair	1	60	0.00 (−2.79, 2.79)	1.000	—	—		
**Analysis and subgroup results of Bergamot supplementation on SBP**								
Overall effect	3	150	−2.09 (−3.33, −0.86)	**0.001**	0.869	0.0%		0.0
Health status								
Metabolic Syndrome	2	90	−2.00 (−3.28, −0.72)	**0.002**	1.000	—	0.596	
Dyslipidemia/Hyperlipidemia	1	60	−3.30 (−7.93, 1.33)	0.162	—	—		
Duration (weeks)								
≤ 12	2	90	−2.00 (−3.28, −0.72)	**0.002**	1.000	—	0.596	
> 12	1	60	−3.30 (−7.93, 1.33)	0.162	—	—		
Intervention type								
Tablet	2	90	−2.00 (−3.28, −0.72)	**0.002**	1.000	—	0.596	
Capsule	1	60	−3.30 (−7.93, 1.33)	0.162	—	—		
Sample size								
< 50	2	90	−2.00 (−3.28, −0.72)	**0.002**	1.000	—	0.596	
≥ 50	1	60	−3.30 (−7.93, 1.33)	0.162	—	—		
Quality of study								
Poor	2	90	−2.00 (−3.28, −0.72)	**0.002**	1.000	—	0.596	
Fair	1	60	−3.30 (−7.93, 1.33)	0.162	—	—		
**Analysis and subgroup results of Bergamot supplementation on Glucose level**								
Overall effect	12	555	−12.12 (−17.05, −7.19)	**< 0.001**	< 0.001	98.7%		72.158
Health status								
Metabolic Syndrome	5	214	−8.61 (−16.60, −0.63)	**0.035**	< 0.001	97.5%	0.175	
Dyslipidemia/Hyperlipidemia	7	341	−15.07 (−19.88, −10.26)	**< 0.001**	< 0.001	98.2%		
Country								
Italy	11	457	−12.71 (−17.80, −7.62)	**< 0.001**	< 0.001	98.8%	0.087	
China	1	98	−4.50 (−12.41, 3.41)	0.265	—	—		
Intervention Age (years)								
< 55	6	290	−9.64 (−16.80, −2.48)	**0.008**	< 0.001	99.3%	< 0.001	
≥ 55	4	206	−9.64 (−18.74, −0.54)	**0.038**	< 0.001	95.4%		
NR	2	59	−24.47 (−27.66, −21.27)	**< 0.001**	0.246	25.6%		
Baseline BMI (kg/m^2^)								
25 ≤ BMI < 30	8	394	−8.65 (−14.90, −2.40)	**0.007**	< 0.001	99.1%	< 0.001	
BMI ≥ 30	2	102	−13.50 (−28.20, 1.10)	0.071	< 0.001	97.8%		
NR	2	59	−24.50 (−27.70, −21.30)	**< 0.001**	0.246	25.6%		
Duration (weeks)								
≤ 12	10	415	−13.68 (−18.77, −8.59)	**< 0.001**	< 0.001	98.7%	0.003	
> 12	2	140	−4.70 (−7.62, −1.79)	**0.002**	0.264	19.7%		
Intervention type								
Tablet	8	353	−12.68 (−18.75, −6.62)	**< 0.001**	< 0.001	99.1%	0.744	
Capsule	4	202	−10.99 (−19.10, −2.89)	**0.008**	< 0.001	94.9%		
Formulations								
Bergamot	8	451	−7.88 (−15.12, −0.46)	**0.037**	< 0.001	96.7%	0.004	
Bergamot Polyphenols	4	104	−20.37 (−24.80, −15.94)	**< 0.001**	< 0.001	98.3%		
Dose (mg/day)								
< 800	8	410	−9.86 (−19.46, −0.27)	**0.044**	< 0.001	99.1%	0.258	
≥ 800	4	145	−16.61 (−23.29, −9.92)	**< 0.001**	< 0.001	96.0%		
Control type								
Placebo	11	475	−12.70 (−17.73, −7.67)	**< 0.001**	< 0.001	98.7%	0.031	
Hypocaloric personalized diet	1	80	−6.00 (−9.40, −2.60)	**0.001**	—	—		
Sample size								
< 50	8	253	−16.48 (−21.81, −11.16)	**< 0.001**	< 0.001	98.9%	< 0.001	
≥ 50	4	302	−2.95 (−6.61, 0.72)	0.115	0.075	56.5%		
Quality of study								
Poor	8	372	−11.94 (−17.70, −6.18)	**< 0.001**	< 0.001	99.0%	< 0.001	
Fair	3	119	−17.32 (−31.31, −3.32)	**0.015**	< 0.001	97.4%		
Good	1	64	2.55 (−2.92, 8.02)	0.361	—	—		
**Analysis and subgroup results of Bergamot supplementation on HOMA‐IR**								
Overall effect	5	198	−0.38 (−0.65, −0.11)	**0.005**	0.004	73.8%		0.067
Health status								
Metabolic Syndrome	4	134	−0.44 (−0.73, −0.16)	**0.003**	0.007	75.3%	0.151	
Dyslipidemia/Hyperlipidemia	1	64	−0.08 (−0.48, 0.32)	0.697	—	—		
Intervention age (years)								
< 55	2	90	−0.25 (−0.47, −0.04)	**0.020**	0.647	0.0%	0.390	
≥ 55	3	108	−0.46 (−0.88, −0.04)	**0.032**	0.004	81.9%		
Baseline BMI (kg/m^2^)								
25 ≤ BMI < 30	4	176	−0.27 (−0.43, −0.12)	**0.001**	0.594	0.0%	< 0.001	
BMI ≥ 30	1	22	−0.84 (−1.10, −0.58)	**< 0.001**	—	—		
Intervention type								
Tablet	3	154	−0.22 (−0.40, −0.03)	**0.025**	0.681	0.0%	0.089	
Capsule	2	44	−0.62 (−1.06, −0.19)	**0.005**	0.025	80.1%		
Formulations								
Bergamot	3	154	−0.22 (−0.40, −0.03)	**0.025**	0.681	0.0%	0.089	
Bergamot Polyphenols	2	44	−0.62 (−1.06, −0.19)	**0.005**	0.025	80.1%		
Dose (mg/day)								
< 800	3	112	−0.31 (−0.48, −0.14)	**< 0.001**	0.650	0.0%	0.664	
≥ 800	2	86	−0.48 (−1.22, 0.27)	0.210	0.002	89.6%		
Sample size								
< 50	4	134	−0.44 (−0.73, −0.16)	**0.003**	0.007	75.3%	0.151	
≥ 50	1	64	−0.08 (−0.48, 0.32)	0.697	—	—		
Quality of study								
Poor	4	134	−0.44 (−0.73, −0.16)	**0.003**	0.007	75.3%	0.151	
Good	1	64	−0.08 (−0.48, 0.32)	0.697	—	—		
**Analysis and subgroup results of Bergamot supplementation on Insulin**								
Overall effect	3	108	−2.93 (−5.06, −0.79)	**0.007**	0.002	84.0%		2.989
Health status								
Metabolic Syndrome	2	44	−3.87 (−5.83, −1.91)	**< 0.001**	0.044	75.2%	0.023	
Dyslipidemia/Hyperlipidemia	1	64	−0.86 (−2.58, 0.86)	0.326	—	—		
Baseline BMI (kg/m^2^)								
25 ≤ BMI < 30	2	86	−1.96 (−3.95, 0.03)	0.054	0.063	71.1%	0.020	
BMI ≥ 30	1	22	−4.90 (−6.36, −3.44)	**< 0.001**	—	—		
NR	0	0	—	—	—	—		
Intervention type								
Tablet	1	64	−0.86 (−2.58, 0.86)	0.326	—	—	0.023	
Capsule	2	44	−3.87 (−5.83, −1.91)	**< 0.001**	0.044	75.2%		
Formulations								
Bergamot	1	64	−0.86 (−2.58, 0.86)	0.326	—	—	0.023	
Bergamot Polyphenols	2	44	−3.87 (−5.83, −1.91)	**< 0.001**	0.044	75.2%		
Dose (mg/day)								
< 800	1	22	−2.90 (−4.20, −1.60)	**< 0.001**	—	—	0.998	
≥ 800	2	86	−2.91 (−6.87, 1.05)	0.150	< 0.001	91.9%		
Sample size								
< 50	2	44	−3.87 (−5.83, −1.91)	**< 0.001**	0.044	75.2%	0.023	
≥ 50	1	64	−0.86 (−2.58, 0.86)	0.326	—	—		
Quality of study								
Poor	2	44	−3.87 (−5.83, −1.91)	**< 0.001**	0.044	75.2%	0.023	
Good	1	64	−0.86 (−2.58, 0.86)	0.326	—	—		
**Analysis and subgroup results of Bergamot supplementation on HDL‐C**								
Overall effect	14	615	5.63 (3.75, 7.51)	**< 0.001**	< 0.001	92.9%		11.298
Health status								
Metabolic Syndrome	5	214	5.93 (2.79, 9.08)	**< 0.001**	< 0.001	94.6%	0.803	
Dyslipidemia/Hyperlipidemia	9	401	5.42 (2.91, 7.93)	**< 0.001**	< 0.001	92.5%		
Country								
Italy	13	517	6.05 (4.17, 7.93)	**< 0.001**	< 0.001	92.8%	< 0.001	
China	1	98	0.00 (−2.83, 2.83)	1.000	—	—		
Intervention Age (years)								
< 55	6	280	4.44 (2.77, 6.11)	**< 0.001**	0.001	75.1%	0.051	
≥ 55	4	206	3.69 (−1.29, 8.68)	0.146	< 0.001	95.2%		
NR	4	129	8.60 (5.56, 11.65)	**< 0.001**	< 0.001	92.7%		
Baseline BMI (kg/m^2^)								
25 ≤ BMI < 30	8	424	3.17 (2.28, 4.07)	**< 0.001**	0.160	33.5%	< 0.001	
BMI ≥ 30	2	102	9.70 (8.52, 10.89)	**< 0.001**	0.446	0.0%		
NR	4	89	8.60 (5.56, 11.65)	**< 0.001**	< 0.001	92.7%		
Duration (weeks)								
≤ 12	12	475	5.47 (3.48, 7.45)	**< 0.001**	< 0.001	93.7%	0.794	
> 12	2	140	6.65 (−2.07, 15.39)	0.135	0.002	89.1%		
Intervention type								
Tablet	8	353	5.70 (3.27, 8.13)	**< 0.001**	< 0.001	92.4%	0.924	
Capsule	6	262	5.51 (2.46, 8.56)	**< 0.001**	< 0.001	93.6%		
Formulations								
Bergamot	8	451	5.05 (2.15, 7.94)	**0.001**	< 0.001	93.1%	0.532	
Bergamot Polyphenols	6	164	6.28 (3.74, 8.82)	**< 0.001**	< 0.001	93.2%		
Dose (mg/day)								
< 800	8	410	4.08 (2.60, 5.56)	**< 0.001**	< 0.001	77.1%	0.026	
≥ 800	6	205	7.63 (4.87, 10.40)	**< 0.001**	< 0.001	92.8%		
Control type								
Placebo	12	505	4.86 (2.90, 6.82)	**< 0.001**	< 0.001	92.7%	< 0.001	
Hypocaloric personalized diet	1	80	11.00 (7.46, 14.54)	**< 0.001**	—	—		
Rosuvastatin	1	30	10.00 (8.35, 11.64)	**< 0.001**	—	—		
Sample size								
< 50	10	313	6.20 (4.14, 8.25)	**< 0.001**	< 0.001	94.1%	0.412	
≥ 50	4	302	3.70 (−1.88, 9.29)	0.194	< 0.001	87.5%		
Quality of study								
Poor	10	422	5.52 (3.46, 7.57)	**< 0.001**	< 0.001	93.0%	0.359	
Fair	3	129	6.76 (1.05, 12.48)	**0.020**	< 0.001	95.2%		
Good	1	64	1.48 (−4.16, 7.12)	0.607	—	—		
**Analysis and subgroup results of Bergamot supplementation on LDL‐C**								
Overall effect	14	615	−39.53 (−53.46, −25.60)	**< 0.001**	< 0.001	99.6%		696.349
Health status								
Metabolic Syndrome	5	214	−36.72 (−61.02, −12.42)	**0.003**	< 0.001	99.4%	0.773	
Dyslipidemia/Hyperlipidemia	9	401	−41.11 (−58.42, −23.81)	**< 0.001**	< 0.001	99.6%		
Country								
Italy	13	517	−42.23 (−56.50, −27.95)	**< 0.001**	< 0.001	99.6%	< 0.001	
China	1	98	−4.24 (−11.87, 3.39)	0.276	—	—		
Intervention Age (years)								
< 55	6	280	−31.51 (−48.23, −14.78)	**< 0.001**	< 0.001	99.2%	0.508	
≥ 55	4	206	−36.74 (−64.39, −9.09)	**0.009**	< 0.001	98.9%		
NR	4	129	−54.62 (−89.83, −19.40)	**0.002**	< 0.001	99.8%		
Baseline BMI (kg/m^2^)								
25 ≤ BMI < 30	8	390	−29.58 (−44.21, −14.95)	**< 0.001**	< 0.001	99.1%	0.328	
BMI ≥ 30	2	102	−49.81 (−90.42, −9.20)	**0.016**	< 0.001	99.0%		
NR	4	123	−54.62 (−89.83, −19.40)	**0.002**	< 0.001	99.8%		
Duration (weeks)								
≤ 12 weeks	12	475	−41.72 (−56.87, −26.57)	**< 0.001**	< 0.001	99.6%	0.071	
> 12 weeks	2	140	−26.91 (−32.34, −21.48)	**< 0.001**	0.337	0.0%		
Intervention type								
Tablet	8	353	−37.40 (−51.95, −22.85)	**< 0.001**	< 0.001	98.9%	0.751	
Capsule	6	262	−42.18 (−67.94, −16.42)	**0.001**	0.001	99.8%		
Formulations								
Bergamot	8	451	−27.59 (−40.18, −15.00)	**< 0.001**	< 0.001	96.8%	0.022	
Bergamot Polyphenols	6	164	−54.75 (−74.22, −35.28)	**< 0.001**	< 0.001	99.8%		
Dose (mg/day)								
< 800	8	410	−30.75 (−46.61, −14.90)	**< 0.001**	< 0.001	98.9%	0.150	
≥ 800	6	205	−51.20 (−74.10, −28.31)	**< 0.001**	< 0.001	99.8%		
Control type								
Placebo	12	505	−41.64 (−56.35, −26.93)	**< 0.001**	< 0.001	99.5%	0.052	
Hypocaloric personalized diet	1	80	−29.00 (−35.91, −22.09)	**< 0.001**	—	—		
Rosuvastatin	1	30	−25.00 (−26.72, −23.28)	**< 0.001**	—	—		
Sample size								
< 50	10	313	−47.63 (−63.98, −31.28)	**< 0.001**	< 0.001	99.7%	0.005	
≥ 50	4	302	−19.04 (−30.21, −7.87)	**0.001**	< 0.001	87.2%		
Quality of study								
Poor	10	422	−39.46 (−56.02, −22.90)	**< 0.001**	< 0.001	99.7%	0.026	
Fair	3	129	−46.49 (−71.48, −21.50)	**< 0.001**	< 0.001	94.1%		
Good	1	64	−19.36 (−28.24, −10.48)	**< 0.001**	—	—		
**Analysis and subgroup results of Bergamot supplementation on TC**								
Overall effect	14	615	−44.69 (−62.00, −27.39)	**< 0.001**	< 0.001	99.5%		1100
Health status								
Metabolic Syndrome	5	214	−39.57 (−62.98, −16.16)	**0.001**	< 0.001	98.8%	0.639	
Dyslipidemia/Hyperlipidemia	9	401	−47.56 (−71.41, −23.72)	**< 0.001**	< 0.001	99.7%		
Country								
Italy	13	517	−47.70 (−65.58, −29.81)	**< 0.001**	< 0.001	99.5%	< 0.001	
China	1	98	−5.41 (−13.91, 3.09)	0.212	—	—		
Intervention Age (years)								
< 55	6	280	−34.16 (−51.03, −17.29)	**< 0.001**	< 0.001	97.7%	0.407	
≥ 55	4	206	−40.95 (−69.14, −12.76)	**0.004**	< 0.001	98.6%		
NR	4	129	−64.48 (−105.74, −23.22)	**0.002**	< 0.001	99.9%		
Baseline BMI (kg/m^2^)								
25 ≤ BMI < 30	8	390	−33.76 (−49.41, −18.10)	**< 0.001**	< 0.001	97.9%	0.348	
BMI ≥ 30	2	102	−49.38 (−93.16, −5.60)	**0.027**	< 0.001	99.0%		
NR	4	123	−64.48 (−105.74, −23.22)	**0.002**	< 0.001	99.9%		
Duration (weeks)								
≤ 12	12	475	−47.90 (−66.97, −28.83)	**< 0.001**	< 0.001	99.6%	0.031	
> 12	2	140	−25.98 (−31.71, −20.25)	**< 0.001**	0.595	0.0%		
Intervention type								
Tablet	8	353	−44.41 (−60.24, −28.59)	**< 0.001**	< 0.001	97.7%	0.984	
Capsule	6	262	−44.76 (−75.35, −14.17)	**0.004**	< 0.001	99.8%		
Formulations								
Bergamot	8	451	−33.54 (−47.76, −19.32)	**< 0.001**	< 0.001	96.5%	0.115	
Bergamot Polyphenols	6	164	−58.97 (−87.20, −30.73)	**< 0.001**	< 0.001	99.8%		
Dose (mg/day)								
< 800	8	410	−35.36 (−51.52, −19.20)	**< 0.001**	< 0.001	97.7%	0.210	
≥ 800	6	205	−57.08 (−86.94, −27.22)	**< 0.001**	< 0.001	99.8%		
Control type								
Placebo	12	505	−48.00 (−65.85, −30.14)	**< 0.001**	< 0.001	99.2%	0.014	
Hypocaloric personalized diet	1	80	−27.00 (−33.86, −20.14)	**< 0.001**	—	—		
Rosuvastatin	1	30	−23.00 (−24.72, −21.27)	**< 0.001**	—	—		
Sample size								
< 50	10	313	−54.32 (−75.45, −33.19)	**< 0.001**	< 0.001	99.6%	0.005	
≥ 50	4	302	−20.66 (−30.43, −10.89)	**< 0.001**	< 0.001	83.4%		
Quality of study								
Poor	10	422	−42.57 (−63.58, −21.57)	**< 0.001**	< 0.001	99.6%	0.081	
Fair	3	129	−58.23 (−93.19, −23.26)	**0.001**	< 0.001	96.6%		
Good	1	64	−26.02 (−32.79, −19.26)	**< 0.001**	—	—		
**Analysis and subgroup results of Bergamot supplementation on TG**								
Overall effect	14	615	−52.47 (−66.09, −38.86)	**< 0.001**	< 0.001	99.1%		633.617
Health status								
Metabolic Syndrome	5	214	−39.38 (−68.58, −10.18)	**0.008**	< 0.001	99.1%	0.17	
Dyslipidemia/Hyperlipidemia	9	401	−61.15 (−73.68, −48.64)	**< 0.001**	< 0.001	98.3%		
Country								
Italy	13	517	−53.75 (−67.78, −39.71)	**< 0.001**	< 0.001	99.1%	0.165	
China	1	98	−31.88 (−59.36, −4.40)	**0.023**	—	—		
Intervention Age (years)								
< 55	6	280	−38.61 (−69.78, −7.43)	**0.015**	< 0.001	99.5%	0.033	
≥ 55	4	206	−45.79 (−71.29, −20.29)	**< 0.001**	< 0.001	96.3%		
NR	4	129	−77.93 (−95.63, −60.24)	**< 0.001**	< 0.001	98.7%		
Baseline BMI (kg/m^2^)								
25 ≤ BMI < 30	8	390	−37.30 (−63.30, −11.30)	**0.005**	< 0.001	99.4%	0.037	
BMI ≥ 30	2	102	−56.36 (−95.58, −17.13)	**0.005**	< 0.001	97.0%		
NR	4	123	−77.93 (−95.63, −60.24)	**< 0.001**	< 0.001	98.7%		
Duration (weeks)								
≤ 12	12	475	−56.51 (−71.15, −41.88)	**< 0.001**	< 0.001	99.2%	0.027	
> 12	2	140	−27.93 (−48.64, −7.23)	**0.008**	0.122	58.1%		
Intervention type								
Tablet	8	353	−52.56 (−79.90, −25.22)	**< 0.001**	< 0.001	99.4%	0.852	
Capsule	6	262	−55.43 (−68.36, −42.51)	**< 0.001**	< 0.001	97.9%		
Formulations								
Bergamot	8	451	−38.32 (−58.49, −18.16)	**< 0.001**	< 0.001	96.8%	0.009	
Bergamot Polyphenols	6	164	−69.63 (−81.61, −57.65)	**< 0.001**	< 0.001	98.7%		
Dose (mg/day)								
< 800	8	410	−41.86 (−68.20, −15.52)	**0.002**	< 0.001	99.1%	0.827	
≥ 800	6	205	−65.67 (−80.44, −50.90)	**< 0.001**	< 0.001	98.8%		
Control type								
Placebo	12	505	−54.14 (−71.22, −37.06)	**< 0.001**	< 0.001	99.1%	0.11	
Hypocaloric personalized diet	1	80	−36.00 (−47.98, −24.02)	**< 0.001**	—	—		
Rosuvastatin	1	30	−48.00 (−50.21, −45.79)	**< 0.001**	—	—		
Sample size								
< 50	10	313	−63.38 (−79.03, −47.73)	**< 0.001**	< 0.001	99.3%	0.001	
≥ 50	4	302	−21.43 (−39.54, −3.33)	0.020	0.009	74.0%		
Quality of study								
Poor	10	422	−51.14 (−66.60, −35.68)	**< 0.001**	< 0.001	99.3%	< 0.001	
Fair	3	129	−73.45 (−125.92, −20.97)	**0.006**	< 0.001	95.3%		
Good	1	64	−4.03 (−19.04, 10.98)	0.599	—	—		
**Analysis and subgroup results of Bergamot supplementation on ALT**								
Overall effect	5	294	−0.20 (−1.43, 1.04)	0.754	0.178	36.5%		0.700
Health status								
Metabolic Syndrome	3	170	−0.19 (−1.07, 0.69)	0.671	0.483	0.0%	0.875	
Dyslipidemia/Hyperlipidemia	2	124	0.33 (−6.04, 6.69)	0.920	0.040	76.2%		
Intervention Age (years)								
< 55	4	230	0.05 (−1.02, 1.13)	0.921	0.307	16.9%	0.089	
≥ 55	1	64	−2.47 (−5.18, 0.24)	0.074	—	—		
Baseline BMI (kg/m^2^)								
25 ≤ BMI < 30	4	214	0.07 (−1.92, 2.05)	0.946	0.112	49.9%	0.620	
BMI ≥ 30	1	80	−0.50 (−1.56, 0.56)	0.353	—	—		
Duration (weeks)								
≤ 12	3	154	−0.36 (−2.25, 1.54)	0.713	0.147	47.9%	0.584	
> 12	2	140	0.93 (−3.24, 5.09)	0.664	0.117	59.2%		
Intervention type								
Tablet	4	234	−0.39 (−1.45, 0.67)	0.468	0.270	23.5%	0.126	
Capsule	1	60	4.1 (−1.56, 9.76)	0.156	—	—		
Dose (mg/day)								
< 800	4	230	0.05 (−1.02, 1.13)	0.921	0.307	16.9%	0.089	
≥ 800	1	64	−2.47 (−5.18, 0.24)	0.074	—	—		
Control type								
Placebo	4	214	0.07 (−1.92, 2.05)	0.946	0.112	49.9%	0.620	
Hypocaloric personalized diet	1	80	−0.50 (−1.56, 0.56)	0.353	—	—		
Sample size								
< 50	2	124	0.49 (−1.07, 2.06)	0.537	0.531	0.0%	0.460	
≥ 50	3	170	−0.55 (−2.85, 1.74)	0.636	0.105	55.7%		
Quality of study								
Poor	3	170	−0.19 (−1.07, 0.69)	0.671	0.483	0.0%	0.089	
Fair	1	60	4.1 (−1.56, 9.76)	0.156	—	—		
Good	1	64	−2.47 (−5.18, 0.24)	0.074	—	—		
**Analysis and subgroup results of Bergamot supplementation on AST**								
Overall effect	5	294	0.13 (−1.48, 1.75)	0.871	0.005	73.2%		2.381
Health status								
Metabolic Syndrome	3	170	−0.06 (−1.82, 1.70)	0.946	0.037	69.8%	0.705	
Dyslipidemia/Hyperlipidemia	2	124	0.99 (−4.15, 6.13)	0.707	0.005	87.3%		
Intervention Age (years)								
< 55	4	230	0.57 (−1.26, 2.40)	0.541	0.013	72.0%	0.116	
≥ 55	1	64	−1.46 (−3.21, 0.29)	0.102	—	—		
Baseline BMI (kg/m^2^)								
25 ≤ BMI < 30	4	214	−0.31 (−2.08, 1.46)	0.732	0.039	64.2%	0.119	
BMI ≥ 30	1	80	1.40 (0.18, 2.62)	**0.024**	—	—		
Duration (weeks)								
≤ 12	3	154	−1.17 (−2.25, −0.10)	**0.032**	0.921	0.0%	0.007	
> 12	2	140	2.12 (−0.04, 4.27)	0.054	0.173	46.2%		
Intervention type								
Tablet	4	234	−0.40 (−1.92, 1.12)	0.603	0.020	69.4%	0.021	
Capsule	1	60	3.8 (0.57, 7.03)	**0.021**	—	—		
Dose (mg/day)								
< 800	4	230	0.57 (−1.26, 2.40)	0.541	0.013	72.0%	0.116	
≥ 800	1	64	−1.46 (−3.21, 0.29)	0.102	—	—		
Control type								
Placebo	4	214	−0.31 (−2.08, 1.46)	0.732	0.039	64.2%	0.119	
Hypocaloric personalized diet	1	80	1.4 (0.18, 2.62)	**0.024**	—	—		
Sample size								
< 50	2	124	−1.00 (−2.37, 0.37)	0.151	1.000	—	0.176	
≥ 50	3	170	0.99 (−1.55, 3.53)	0.445	0.005	81.2%		
Quality of study								
Poor	3	170	−0.06 (−1.82, 1.70)	0.946	0.037	69.8%	0.019	
Fair	1	60	3.8 (0.57, 7.03)	**0.021**	—	—		
Good	1	64	−1.46 (−3.21, 0.29)	0.102	—	—		
**Analysis and subgroup results of Bergamot supplementation on GGT**								
Overall effect	4	214	−0.85 (−4.04, 2.35)	0.605	0.112	50.0%		4.842
Health status								
Metabolic Syndrome	2	90	−0.45 (−5.35, 4.45)	0.858	0.019	81.9%	0.638	
Dyslipidemia/Hyperlipidemia	2	124	−2.20 (−7.65, 3.24)	0.428	0.922	0.0%		
Intervention Age (years)								
< 55	3	150	−0.56 (−4.64, 3.52)	0.786	0.061	64.3%	0.637	
≥ 55	1	64	−2.35 (−8.54, 3.84)	0.457	—	—		
Duration (weeks)								
≤ 12	3	154	−0.84 (−4.54, 2.87)	0.658	0.051	66.4%	0.888	
> 12	1	60	−1.70 (−13.17, 9.77)	0.771	—	—		
Intervention type								
Tablet	3	154	−0.84 (−4.54, 2.87)	0.658	0.051	66.4%	0.888	
Capsule	1	60	−1.70 (−13.17, 9.77)	0.771	—	—		
Dose (mg/day)								
< 800	3	150	−0.56 (−4.64, 3.52)	0.786	0.061	64.3%	0.637	
≥ 800	1	64	−2.35 (−8.54, 3.84)	0.457	—	—		
Sample size								
< 50	2	90	−0.45 (−5.35, 4.45)	0.858	0.019	81.9%	0.638	
≥ 50	2	124	−2.20 (−7.65, 3.24)	0.428	0.922	0.0%		
Quality of study								
Poor	2	90	−0.45 (−5.35, 4.45)	0.858	0.019	81.9%	0.891	
Fair	1	60	−1.70 (−13.17, 9.77)	0.771	—	—		
Good	1	64	−2.35 (−8.54, 3.84)	0.457	—	—		

*Note:* Bold values indicate statistically significant results (*p* < 0.05).

Abbreviations: ALT, Alanine Aminotransferase; AST, Aspartate Aminotransferase; BMI, Body Mass Index; DBP, Diastolic Blood Pressure; GGT, Gamma‐Glutamyl Transferase; HDL‐C, High‐Density Lipoprotein Cholesterol; HOMA‐IR, Homeostatic Model Assessment of Insulin Resistance; LDL‐C, Low‐Density Lipoprotein Cholesterol; SBP, Systolic Blood Pressure; TC, Total Cholesterol; TG, Triglycerides; WC, Waist Circumference; WMD, weighted mean difference.

WC, SBP, HOMA‐IR, and insulin showed statistically significant pooled effects among participants with metabolic syndrome, while glucose, HDL‐C, LDL‐C, TC, and TG showed significant pooled effects in both health‐status groups.

SBP, glucose, HOMA‐IR, insulin, HDL‐C, LDL‐C, and TC showed statistically significant pooled effects among studies conducted in Italy, while TG showed significant pooled effects in both Italy and China.

BMI showed statistically significant pooled effects among participants aged ≥ 55 years, while WC and SBP showed significant pooled effects among those aged < 55 years. HOMA‐IR showed significant pooled effects in both age groups, whereas HDL‐C showed significant pooled effects among participants aged < 55 years and those with not reported age. Glucose, LDL‐C, TC, and TG showed significant pooled effects across both age groups and studies with not reported age.

WC showed a statistically significant pooled effect among participants with a baseline BMI of 25 ≤ BMI < 30, while SBP, insulin, and AST showed significant pooled effects among those with a baseline BMI ≥ 30. HOMA‐IR showed significant pooled effects in both baseline BMI groups (25 ≤ BMI < 30 and BMI ≥ 30). Glucose showed a significant pooled effect among participants with a baseline BMI of 25 ≤ BMI < 30 and those with not reported BMI. HDL‐C, LDL‐C, TC, and TG showed significant pooled effects across both baseline BMI groups and studies with not reported BMI.

WC, SBP, HOMA‐IR, insulin, HDL‐C, and AST showed statistically significant pooled effects among interventions lasting ≤ 12 weeks, while glucose, LDL‐C, TC, and TG showed significant pooled effects across both duration groups. SBP showed a statistically significant pooled effect among studies using tablet formulations, while BMI, insulin, and AST showed significant pooled effects among studies using capsule formulations. Glucose, HOMA‐IR, HDL‐C, LDL‐C, TC, and TG showed significant pooled effects across both intervention types. BMI and insulin showed statistically significant pooled effects among studies using Bergamot polyphenols, while WC, weight, and SBP showed significant pooled effects among studies using Bergamot. Glucose, HOMA‐IR, HDL‐C, LDL‐C, TC, and TG showed significant pooled effects across both intervention types. WC, SBP, HOMA‐IR, and insulin showed statistically significant pooled effects among studies using doses < 800 mg/day, while BMI showed a significant pooled effect among studies using doses ≥ 800 mg/day. Glucose, HDL‐C, LDL‐C, TC, and TG showed significant pooled effects across both dosage groups (< 800 and ≥ 800 mg/day). WC, SBP, HOMA‐IR, and insulin showed statistically significant pooled effects among studies using placebo as the control, while AST showed a significant pooled effect among studies using a hypocaloric personalized diet as the control. Glucose showed a significant pooled effect across both placebo and hypocaloric personalized diet control groups. HDL‐C, LDL‐C, TC, and TG showed significant pooled effects across all control types (placebo, hypocaloric personalized diet, and rosuvastatin). WC, weight, SBP, glucose, HOMA‐IR, insulin, HDL‐C, and TG showed statistically significant pooled effects among studies with a sample size < 50, while LDL‐C and TC showed significant pooled effects across both sample‐size groups. WC, SBP, HOMA‐IR, and insulin showed statistically significant pooled effects among studies with poor quality, while AST showed a significant pooled effect among studies with fair quality. Glucose, HDL‐C, and TG showed significant pooled effects across studies with poor and fair quality. LDL‐C and TC showed significant pooled effects across all study‐quality categories.

### 3.10 Meta‐Regression and Non‐Linear Dose–Response Analysis

Figures S1 and S2 present a comprehensive meta‐regression analysis evaluating the impact of bergamot dosage and intervention duration on cardiovascular risk factors. Using a regression model, we aimed to elucidate the complex relationship between bergamot supplementation and cardiovascular outcomes. The meta‐regression analysis revealed significant associations between bergamot dosage and multiple outcomes, including BMI (*p* = 0.011), indicating that higher doses may improve BMI. Additionally, meta‐regression revealed that intervention duration had significant associations with glucose (*p* = 0.014) and TG (*p* = 0.046), suggesting that longer durations may positively affect glucose and TG.

The dose–response analysis showed significant associations between duration of intervention and glucose level (*p* = 0.011) and TG (*p* = 0.009). For glucose, the dose–response relationship appeared to be non‐linear, with the largest improvement observed at approximately 4 weeks of intervention. However, the dosage of bergamot did not show any significant association with GLUCOSE and TG. In addition, dose–response analysis showed significant associations between dose and duration of Bergamot supplementation with HDL level (*p* = 0.030, *p* = 0.038). There was significant association between duration of intervention and AST (*p* < 0.001) (Figures S3 and S4).

### 3.11 GRADE Assessment

The GRADE profile of the certainty of evidence for the outcomes of bergamot supplementation is presented in Table 4. The certainty of evidence was rated as very low for BMI, weight, glucose, HOMA‐IR, insulin, GGT, HDL‐C, LDL‐C, TC, TG, DBP, SBP ALT and AST. These outcomes were assessed as having “very low” certainty, with important limitations related to risk of bias, inconsistency, indirectness. The certainty of evidence was rated as low for WC.

**TABLE 4:** GRADE profile of Bergamot supplementation on cardiovascular risk factors.

Outcomes	Risk of bias	Inconsistency	Indirectness	Imprecision	Publication bias	Number INT/CON	WMD (95% CI)	Quality of evidence
BMI	Serious^a^	Very serious limitations^b^	Not serious	Serious^d^	Not serious	178/134	−1.01 (−2.10, 0.08)	⨁◯◯◯ Very low
WC	Serious^a^	Not serious	Not serious	Not serious	Serious^e^	181/151	−0.96 (−1.91, −0.01)	⨁⨁◯◯ Low
Weight	Serious^a^	Serious^f^	Not serious	Serious^d^	Not serious	181/165	−2.23 (−4.90, 0.43)	⨁◯◯◯ Very low
DBP	Serious^a^	Very serious limitations^b^	Serious limitations^c^	Very serious^d^	Not serious	90/60	−0.37 (−2.71, 1.98)	⨁◯◯◯ Very low
SBP	Serious^a^	Not serious	Serious limitations^c^	Not serious	Serious^e^	90/60	−2.09 (−3.33, −0.86)	⨁◯◯◯ Very low
Glucose	Serious^a^	Very serious limitations^b^	Not serious	Not serious	Serious^e^	320/235	−12.12 (−17.05, −7.19)	⨁◯◯◯ Very low
HOMA‐IR	Serious^a^	Serious^f^	Serious limitations^c^	Not serious	Not serious	123/75	−0.38 (−0.65, −0.11)	⨁◯◯◯ Very low
Insulin	Serious^a^	Very serious limitations^b^	Serious limitations^c^	Not serious	Not serious	63/45	−2.93 (−5.06, −0.79)	⨁◯◯◯ Very low
HDL‐C	Serious^a^	Very serious limitations^b^	Not serious	Not serious	Not serious	350/265	5.63 (3.75, 7.51)	⨁◯◯◯ Very low
LDL‐C	Serious^a^	Very serious limitations^b^	Not serious	Not serious	Not serious	350/265	−39.53 (−53.46, −25.60)	⨁◯◯◯ Very low
TC	Serious^a^	Very serious limitations^b^	Not serious	Not serious	Not serious	350/265	−44.69 (−62.00, −27.39)	⨁◯◯◯ Very low
TG	Serious^a^	Very serious limitations^b^	Not serious	Not serious	Not serious	350/265	−52.47 (−66.09, −38.86)	⨁◯◯◯ Very low
ALT	Serious^a^	Not serious	Serious limitations^c^	Serious^d^	Not serious	163/131	−0.20 (−1.43, 1.04)	⨁◯◯◯ Very low
AST	Serious^a^	Serious^f^	Serious limitations^c^	Serious^d^	Not serious	163/131	0.13 (−1.48, 1.75)	⨁◯◯◯ Very low
GGT	Serious^a^	Serious^f^	Serious limitations^c^	Serious^d^	Not serious	123/91	−0.85 (−4.04, 2.35)	⨁◯◯◯ Very low

Abbreviations: ALT, Alanine Aminotransferase; AST, Aspartate Aminotransferase; BMI, Body Mass Index; CI, Confidence Interval; CON, Control Group; DBP, Diastolic Blood Pressure; GGT, Gamma‐Glutamyl Transferase; HDL‐C, High‐Density Lipoprotein Cholesterol; HOMA‐IR, Homeostatic Model Assessment of Insulin Resistance; INT, Intervention Group; LDL‐C, Low‐Density Lipoprotein Cholesterol; SBP, Systolic Blood Pressure; TC, Total Cholesterol; TG, Triglycerides; WC, Waist Circumference; WMD, Weighted Mean Difference.

^a^ More than 20% of RCTs for this outcome had a high risk of bias for at least one component of the Cochrane risk of bias tool. Those biases had a significant effect on the results of RCTs.

^b^ The *I* ^2^ value was > 75% (or Heterogeneity among the studies was high).

^c^ Downgraded for indirectness in country.

^d^ Downgraded since the 95% CI crosses the threshold of interest.

^e^ Potential small‐study effects were suggested by Egger's test (*p* < 0.05).

^f^ The *I* ^2^ value was ≥ 50% (or Heterogeneity among the studies was moderate).

## 4 Discussion

Bergamot, due to its unique phytochemical composition, particularly flavonoids and flavonoid glycosides has recently attracted scientific interest for its traditional use in improving cardiovascular health and this study aimed to assess the effects of bergamot on cardiovascular outcomes in adults (Formisano et al. 2019). This meta‐analysis of nine studies including 615 participants demonstrated that Bergamot was associated with statistically significant changes in several cardiovascular surrogate outcomes, including anthropometric measurements, blood pressure, glycemic profile, and lipid profile.

Regarding anthropometric outcomes, this study showed that bergamot supplementation significantly reduced WC but modest associations with body weight or BMI. Previous clinical studies either did not assess weight or BMI (Gliozzi et al. 2013; Mollace et al. 2011; Mollace et al. 2019) or reported no significant changes in BMI (Gliozzi et al. 2014). In addition, the pooled reduction of WC was < 1 cm, and its clinical importance is uncertain and likely modest (Hosseinpanah et al. 2010). In contrast, animal studies in rodents have observed alterations in BMI, although these effects were modest, with only an 8% reduction in weight gain after 14 weeks of bergamot treatment. The limited effect in these models may be explained by the use of an extremely obesogenic cafeteria diet to induce metabolic syndrome and non‐alcoholic steatohepatitis. Consistent with previous trials, modest improvements in WC and body fat percentage were observed with high‐dose bergamot phytocomplex supplementation; however, those limited changes were likely influenced by small sample sizes, normal baseline values, heterogeneity among patients with metabolic syndrome, and diverse genetic and environmental factors such as diet and physical inactivity (Cai et al. 2017) and our study demonstrated a more significant reduction in WC, suggesting that under more robust conditions, bergamot supplementation may exert stronger anthropometric benefits.

The pooled analyzes showed statistically significant changes in LDL‐C, TC, TG, HDL‐C, glucose, insulin, and HOMA‐IR. However, heterogeneity was extreme for glucose and all lipid outcomes (*I* ^2^ = 92.9%–99.6%), indicating that the magnitude of the pooled estimates varied substantially across studies and should not be interpreted as a uniform treatment effect. These findings are similar to previous studies regarding bergamot's favorable effects on cardiometabolic risk factors (Mollace et al. 2019; Formisano et al. 2019).

Formisano et al. (2019) indicated that these beneficial effects may be attributed to the unique phytochemical characteristics of bergamot phytosome, which contains approximately 40 polyphenolic components such as naringin, neoeriocitrin, neohesperidin, brutieridin, and melitidin comprising at least 45% of the extract (Formisano et al. 2019). A key mechanism may involve activation of adenosine monophosphate‐activated protein kinase (AMPK). As an energy sensor, AMPK regulates glucose and lipid metabolism to maintain ATP levels. Once activated, AMPK improves insulin sensitivity and consequently enhances glucose uptake in muscle cells to make ATP through glycolysis. In addition, AMPK inhibits anabolic pathways including cholesterol biosynthesis (via downregulation of 3‐hydroxy‐3‐methylglutaryl‐CoA reductase), fatty acid synthesis (through inhibition of acetyl‐CoA carboxylase), and protein synthesis (Rondanelli et al. 2021). The hypolipidemic and hypoglycemic effects of bergamot are supported by flavanones such as naringin and naringenin that can upregulate AMPK activity (Zygmunt et al. 2010). This mechanism may also improve insulin sensitivity and glucose homeostasis, which may explain the improvements in insulin and HOMA‐IR levels observed in our study. These mechanisms provide biological plausibility only and cannot explain or validate the unusually large pooled lipid estimates.

Our findings align with those of Mollace et al. (2019), who emphasized bergamot's hypolipidemic and hypoglycemic properties in human trials (Mollace et al. 2019). The findings of this study are consistent with prior investigations regarding the potential effects of bergamot phytosome on lipid and glycemic outcomes.

In contrast, our study showed no significant effects on ALT, AST and GGT. However, previous studies in animal and human studies, have shown significant reductions in ALT and AST after bergamot supplementation, particularly among individuals with non‐alcoholic fatty liver disease or hyperlipidemia (Gliozzi et al. 2014; Ferro et al. 2020; Musolino et al. 2020). A likely explanation for this discrepancy is that the included studies in our analysis involved patients with metabolic disorders or dyslipidemia but without underlying liver conditions.

Moreover, previous studies found that weight loss can be associated with ALT reductions due to improved hepatic lipid mobilization; since no significant weight changes occurred in the pooled participants, this may explain the lack of effect on liver enzymes (Ferro et al. 2020; Suzuki et al. 2005). Another contributing factor may be the small sample size.

Bergamot intervention also resulted in a significant decrease in SBP in our study. This is consistent with findings from Spina et al. (2024) study in humans and Alam et al. (2013) in animals, which also reported blood pressure‐lowering effects of bergamot (Spina et al. 2024; Alam et al. 2013). Pre‐clinical studies suggest that these blood pressure‐lowering effects may be attributed to the rich content of flavonoids in bergamot including naringin, hesperidin, and naringenin (Alam et al. 2013; Baron et al. 2021). Flavonoids improve bioavailability of nitric oxide, and reduce oxidative stress and inflammatory responses, promoting vasodilation and lowering blood pressure. Moreover, dietary flavones play a role in preserving endothelium‐dependent vasodilation, supporting normal blood flow and improving both SBP and DBP (Dohadwala et al. 2011; Ikemura et al. 2012). However, the pooled reduction was approximately 2.09 mmHg and should be regarded as modest at the individual level.

### 4.1 Clinical Implications

The results of this meta‐analysis offer important clinical information for healthcare providers managing patients with metabolic syndrome, insulin resistance, and cardiovascular risk factors. The significant reductions in glucose, insulin, and HOMA‐IR suggest possible improvements in glycemic markers, particularly in patients with elevated cardiometabolic risk. However, certainty was very low for all outcomes except WC. Some subgroup analyzes suggested potentially greater effects among participants receiving ≥ 800 mg/day and those aged ≥ 55 years. However, these findings were based on small and potentially underpowered subgroup analyzes and should therefore be considered hypothesis‐generating rather than evidence for an optimal dose or a specific target population. Bergamot supplementation was associated with a reduction in SBP but not DBP. While the absolute change in SBP may appear modest at the individual level, even small reductions in SBP have been shown to yield meaningful reductions in cardiovascular morbidity and mortality across populations (Bundy et al. 2017). These findings align with previous clinical and preclinical studies supporting the blood pressure‐lowering potential of bergamot‐derived flavonoids, particularly hesperidin and naringenin, which may exert vasodilatory and anti‐inflammatory effects via nitric oxide–mediated mechanisms (Cappello et al. 2016; Carpenito et al. 2025). These results suggest that bergamot may offer additional cardiovascular protection, especially for individuals with coexisting hypertension and metabolic dysfunction.

While no significant changes were observed in body weight or BMI across the pooled analysis, a modest but statistically significant reduction in WC highlights bergamot's potential to reduce central adiposity, an important target in cardiometabolic disease management. Central fat accumulation has been closely linked to insulin resistance, systemic inflammation, and adverse lipid profiles. WC reduction was more apparent in studies with shorter intervention durations (≤ 12 weeks), although the subgroup findings should be interpreted cautiously given the limited number of studies.

Bergamot supplementation was associated with significant improvements in lipid profiles, including reductions in LDL‐C, TC, and TG, with an increase in HDL‐C. The large pooled reductions in LDL‐C and TC require cautious interpretation because heterogeneity exceeded 99%, formulations and populations differed, and effects were smaller in studies with larger samples. Although leave‐one‐out analyzes did not identify a single study that fully explained the estimates, small‐study effects, selective reporting, and other biases remain plausible. These findings do not support replacing established lipid‐lowering therapy with bergamot.

Although liver enzyme changes were generally nonsignificant, subgroup analyzes suggested a slight increase in AST among participants with obesity and in studies using capsule formulations. Given the small number of studies contributing to these subgroup analyzes, these findings should be interpreted cautiously and considered hypothesis‐generating. Further well‐designed studies with adequate follow‐up are needed to clarify the potential effects of bergamot supplementation on liver enzymes.

When considering safety, adverse‐event reporting was limited. In Cai et al., two participants receiving CitriCholess withdrew because of dizziness. The available short‐duration evidence is insufficient to establish safety, and one included study noted mild events such as moderate gastric pyrosis in a few individuals receiving bergamot extract and occasional headache or constipation in the placebo group (Cai et al. 2017; Capomolla et al. 2019). Importantly, no clinically relevant changes were observed in hematological parameters or liver enzymes, and neither myalgia nor elevations in serum creatine phosphokinase or transaminases were detected.

Beyond its metabolic effects, bergamot also demonstrates antioxidant and cardioprotective potential in preclinical models. The antioxidant and cardioprotective effects of BPF have been evaluated in the context of cardiac damage induced by doxorubicin (DOXO), a commonly used antineoplastic drug. In animal studies, co‐administration of BPF with DOXO resulted in significant reductions in cardiomyocytic apoptosis and reactive hypertrophy, largely through enhanced autophagic activity. This combination also counteracted DOXO‐induced cardiotoxicity by reducing ROS production and improving the survival of resident endogenous c‐kitpos cardiac stem cells (Carresi et al. 2018). These findings extend the potential clinical relevance of bergamot beyond metabolic health, highlighting its role in cardioprotection in settings of oxidative stress and drug‐induced injury.

Overall, the current evidence is insufficient to integrate bergamot into standard cardiovascular prevention or to recommend it as an alternative to established lifestyle and pharmacological interventions. Any use should be regarded as adjunctive and individualized until larger, well‐conducted trials provide more certain evidence of clinically meaningful benefit and long‐term safety.

### 4.2 Strengths and Limitations

A critical strength of our meta‐analysis is its methodological rigor and adherence to established scientific standards. This review was conducted using a prospectively registered protocol, PRISMA‐based reporting, and comprehensive searches of five major databases and reference lists. The application of the GRADE framework to assess the strength of evidence represents a significant advancement over previous reviews, offering a detailed assessment of the quality and consistency of included studies across different outcomes. Furthermore, random‐effects models, heterogeneity statistics, leave‐one‐out analyzes, study‐quality subgroups, and meta‐regression and dose–response analyzes were used to characterize the available evidence. These procedures improve transparency and allow uncertainty to be examined, but they cannot compensate for limitations in the primary trials.

Compared with the qualitative review by Lamiquiz‐Moneo et al., the present review quantitatively pooled randomized trials and evaluated a broader set of lipid, glycemic, blood‐pressure, anthropometric, and liver outcomes (Lamiquiz‐Moneo et al. 2020). In addition to assessing lipid profiles, we included a wider range of cardiovascular outcomes including lipid profile, blood pressure, glycemic profile and liver function. Moreover, our study incorporated advanced statistical techniques such as meta‐regression, dose–response analysis, and GRADE assessment to evaluate the certainty of evidence. Their search strategy was also limited, covering only four databases and excluding important sources like Web of Science and Scopus. The lack of a detailed search protocol in their methodology further raises concerns about the comprehensiveness of their findings. In contrast, our study adhered to a transparent and rigorous search strategy, strengthening the validity and reliability of our results.

Compared with the meta‐analysis by Sadeghi‐dehsahraei et al., which included five trials searched through November 2021, this review included nine RCTs and more recent evidence (Sadeghi‐dehsahraei et al. 2022). However, the evidence base remained small. Because fewer than 10 independent trials contributed to each outcome, Egger's tests and other publication‐bias procedures had limited power, and the apparent evidence of small‐study effects for WC, SBP, and glucose should be interpreted cautiously. Given the very small number of studies, funnel‐plot procedures, including Egger's test and trim‐and‐fill analysis, have limited reliability; therefore, neither significant nor non‐significant findings can reliably confirm or exclude publication bias.

The findings of the present meta‐analysis regarding bergamot's lipid‐reduction associations are broadly consistent with the conclusions of Osadnik et al., who conducted a network meta‐analysis of 131 RCTs enrolling 13,062 participants and identified bergamot and red yeast rice as the most effective nutraceuticals for LDL‐C and TC reduction, reporting a bergamot‐associated LDL‐C reduction of approximately −46.8 mg/dL. However, several important differences distinguish the two analyzes. Osadnik et al. employed a network meta‐analysis framework allowing indirect comparisons across multiple nutraceuticals, whereas the present study used a direct‐comparison random‐effects meta‐analysis focused exclusively on bergamot supplementation. Furthermore, Osadnik et al. restricted their outcomes to lipid fractions (LDL‐C, TC, HDL‐C, TG), while the present analysis extends the evidence base to include glycemic markers (glucose, HOMA‐IR, insulin), blood pressure, anthropometric measures, and liver enzymes, accompanied by formal GRADE‐based certainty assessment for each outcome. Notably, Osadnik et al. themselves cautioned that the evidence for bergamot was based on relatively small study groups (*n* = 3) and may require further investigation, a concern reinforced by the very high heterogeneity (*I* ^2^ > 90%) and low GRADE certainty ratings observed in the present analysis (Osadnik et al. 2022).

Despite the strengths of our meta‐analysis, several limitations must be acknowledged. The relatively small number of included studies (9 RCTs) and the moderate sample sizes introduce a degree of heterogeneity that could influence the overall results. Even with the use of random‐effects models to address this variability, the possibility of remaining confounding factors cannot be eliminated. Another limitation is that all included trials were conducted in Italy and China, which may limit the generalizability of our findings to other populations with different genetic backgrounds, dietary patterns, and environmental factors. This geographical limitation reflects the current state of the evidence base rather than a methodological choice, highlighting a significant gap in global research on bergamot supplementation that future studies should address.

Furthermore, the included studies assessed surrogate risk factors rather than cardiovascular events, hospitalization, or mortality; consequently, the review cannot establish cardiovascular risk reduction. In addition, the variability in baseline health status among participants in different studies introduces complexity in interpreting results across conditions. Our subgroup analyzes attempted to address this by stratifying results based on health conditions, but larger sample sizes within each health category would provide more definitive conclusions. Finally, despite our comprehensive approach, GRADE certainty was low or very low for all outcomes, indicating that future high‐quality research is needed to strengthen the evidence base for bergamot's cardiometabolic effects.

### 4.3 Future Directions

Future research should prioritize adequately powered, prospectively registered, multicenter RCTs conducted in geographically and ethnically diverse populations. Trials should use chemically characterized and standardized bergamot preparations, prespecified doses and treatment durations, appropriate placebo controls, and consistent monitoring of diet, physical activity, medication use, adherence, and adverse events. These design features are needed to reduce clinical and methodological heterogeneity.

Longer follow‐up is required to determine whether changes in lipid, glycemic, blood‐pressure, or anthropometric measures are sustained and clinically meaningful. Future trials should prespecify minimally important differences and, where feasible, assess cardiovascular events or validated long‐term risk outcomes rather than relying exclusively on short‐term surrogate markers. Multi‐arm dose‐ranging trials would provide stronger evidence about dose–response relationships than comparisons of doses across heterogeneous studies.

Direct comparisons of standardized bergamot formulations and carefully designed adjunctive studies with established therapies may clarify whether formulation influences response and whether any additional benefit is clinically meaningful. Protocols, extracted datasets, analytic code, and complete outcome data should be made publicly available to improve reproducibility and reduce selective reporting.

Mechanistic and omics studies may help identify active compounds and biological pathways, but they should complement rather than substitute for rigorous clinical trials. Collectively, these priorities would allow more reliable evaluation of benefit, harm, dose, duration, and potential treatment‐response modifiers.

## 5 Conclusion

In this systematic review and meta‐analysis of nine RCTs, bergamot supplementation was associated with statistically significant changes in selected cardiometabolic risk factors, including glucose, HOMA‐IR, insulin, LDL‐C, TC, TG, HDL‐C, SBP, and WC. However, high heterogeneity, potential small‐study effects in some outcomes (WC, SBP, glucose), and the predominantly poor quality of included studies limit the certainty and generalizability of these results. GRADE certainty was very low for all outcomes except WC, for which certainty was low. These very low‐certainty associations should not be interpreted as establishing the clinical efficacy of bergamot or as support for replacing standard treatment. Larger, high‐quality RCTs with standardized formulations, longer follow‐up, diverse populations, and clinically meaningful outcomes are required.

## Author Contributions

**Mahla Chambari:** conceptualization, investigation, writing – original draft, writing – review and editing, methodology, formal analysis, data curation, visualization, software. **Helia Mardani:** writing – original draft, methodology, writing – review and editing. **Marjan Roshandel:** writing – original draft, methodology. **Hossein Rafiei:** writing – review and editing, supervision. **Whitney Linsenmeyer:** writing – review and editing, supervision. **Maryam Jafari:** writing – original draft, writing – review and editing, methodology. **Vaidehi Ulaganathan:** writing – review and editing. **Ali Jafari:** conceptualization, investigation, writing – original draft, writing – review and editing, supervision, visualization, data curation.

## Funding

The authors have nothing to report.

## Ethics Statement

The authors have nothing to report.

## Consent

The authors have nothing to report.

## Conflicts of Interest

Ali Jafari holds an editorial position at *Systematic Reviews*. All other authors of this publication declare that they have no affiliations with, or involvement in, any organization or entity with a financial interest (including honoraria, educational grants, participation in speakers' bureaus, memberships, employment, consultancies, stock ownership or other equity interests, expert testimony, or patent‐licensing arrangements) or a non‐financial interest (such as personal or professional relationships, affiliations, knowledge, or beliefs) relevant to the subject matter or materials discussed in this manuscript.

## Supporting information


**Table S1:** Search strategy to find potential eligible randomized controlled trials (January 30, 2025).
**Table S2:** A summary of excluded articles after full text review.
**Figure S1:** Random‐effects meta‐regression plots of the association between bergamot dosage (mg/d) and cardiovascular disease outcomes (a: body mass index, b: waist circumference, c: weight, d: diastolic blood pressure, e: systolic blood pressure, f: glucose, g: homeostatic model assessment of insulin resistance, h: insulin, i: high‐density lipoprotein cholesterol, j: low‐density lipoprotein cholesterol, k: total cholesterol, m: triglycerides, n: alanine aminotransferase, o: aspartate aminotransferase, p: gamma‐glutamyl transferase).
**Figure S2:** Random‐effects meta‐regression plots of the association between duration of intervention and cardiovascular disease outcomes (a: body mass index, b: waist circumference, c: weight, d: diastolic blood pressure, e: systolic blood pressure, f: glucose, g: homeostatic model assessment of insulin resistance, h: insulin, i: high‐density lipoprotein cholesterol, j: low‐density lipoprotein cholesterol, k: total cholesterol, m: triglycerides, n: alanine aminotransferase, o: aspartate aminotransferase, p: gamma‐glutamyl transferase).
**Figure S3:** Non‐linear dose–response relations between bergamot dosage (mg/d) and cardiovascular disease outcome (a: body mass index, b: waist circumference, c: weight, d: diastolic blood pressure, e: systolic blood pressure, f: glucose, g: homeostatic model assessment of insulin resistance, h: insulin, i: high‐density lipoprotein cholesterol, j: low‐density lipoprotein cholesterol, k: total cholesterol, m: triglycerides, n: alanine aminotransferase, o: aspartate aminotransferase, p: gamma‐glutamyl transferase).
**Figure S4:** Non‐linear dose–response relations between duration of intervention and cardiovascular disease outcomes (a: body mass index, b: waist circumference, c: weight, d: diastolic blood pressure, e: systolic blood pressure, f: glucose, g: homeostatic model assessment of insulin resistance, h: insulin, i: high‐density lipoprotein cholesterol, j: low‐density lipoprotein cholesterol, k: total cholesterol, m: triglycerides, n: alanine aminotransferase, o: aspartate aminotransferase, p: gamma‐glutamyl transferase).

**Data S1:** Supporting Information.

## Data Availability

All relevant extracted data are provided in the manuscript and Supporting Information. The primary data are available from the original included studies. The complete extracted dataset and Stata code are available from the corresponding author upon reasonable request.
