# Supplementary material for: Bergamot (Citrus bergamia) Supplementation and Cardiovascular Disease Risk Factors: A Systematic Review and Meta‐Analysis
Source: Food Sci Nutr. 2026 Sep 15;14(9):e72357. doi: 10.1002/fsn3.72357 (PMC13575982; doi:10.1002/fsn3.72357)
Supplement: Supplementary file 2 [file FSN3-14-e72357-s002.docx]

**Bergamot *(Citrus bergamia)* Supplementation and Cardiovascular Disease Risk Factors: A Systematic Review and Meta-Analysis**

**Mahla Chambari^1,2^, Helia Mardani^3^, Marjan Roshandel^4^, Hossein Rafiei^5^, Whitney Linsenmeyer^5^, Maryam Jafari^6^, Vaidehi Ulaganathan^1*^, Ali Jafari^7,8*^**

*^1^Department of Food Science and Nutrition, Faculty of Applied Sciences, UCSI University, 56000 Cheras, Wilayah Persekutuan Kuala Lumpur, Malaysia*

*^2^Faculty of Medicine, Navoi State University, Navoi, Uzbekistan*

*^3^Students' Scientific Research Center (SSRC), Tehran University of Medical Sciences, Tehran, Iran*

*^4^Master student in Medical Surgical Nursing, Student Research Committee, Faculty of Nursing and Midwifery, Mashhad University of Medical Sciences, Mashhad, Iran.
^5^Department of Nutrition and Dietetics, Saint Louis University, Saint Louis, MO, USA*

*^6^Student Research Committee, School of Medicine, Anzali International Campus, Guilan University of Medical Sciences, Rasht, Iran*

*^7^Student Research Committee, Department of Community Nutrition, Faculty of Nutrition Sciences and Food Technology, National Nutrition and Food Technology Research Institute, Shahid Beheshti University of Medical Sciences, Tehran, Iran*

*^8^Systematic Review and Meta-analysis Expert Group (SRMEG), Universal Scientific Education and Research Network (USERN), Tehran, Iran*

Supplementary materials**:** Supplementary Tables 1-2, Supplementary Figure 1-4, and Supplementary References.

| **Supplementary Table 1.** Search strategy to find potential eligible randomised controlled trials (January 30, 2025) | |
| --- | --- |
| **Groups** | **Descriptors** |
| Intervention | "Bergamot" OR "Citrus Bergamia" OR "bergamot essential oil" OR "bergamot juice" OR "bergamot peel" OR "Bergamot Extract" OR "Bergamot Leaves Extract" OR "KoksalGarry" OR "Citrus bergamia Risso et Poiteau" OR "Bergamot Citrus" OR "Citrus bergamias" OR "Bergamot supplementation" OR "Bergamot flavonoids" OR "Citrus Bergamia juice" OR "Citrus Bergamia Extract" |
| Design | Intervention OR “Intervention Study” OR “Intervention Studies” OR “controlled trial” OR random* OR placebo OR “clinical trial” OR Trial OR “randomized controlled trial” OR “randomized clinical trial” OR RCT OR blinded OR “double blind” OR “double blinded” OR trial* OR “Pragmatic Clinical Trial” OR “Cross-Over Studies” OR “Cross-Over” OR “Cross-Over Study” OR parallel OR “parallel study” OR “parallel trial” |

**PubMed**

Number of localized studies: 2,404

|  | **Descriptors** | **Number of studies reached** |
| --- | --- | --- |
| #1 | "Citrus"[Mesh] OR "Citrus"[Title/Abstract] OR "Bergamot"[Title/Abstract] OR "Citrus Bergamia"[Title/Abstract] OR "bergamot essential oil"[Title/Abstract] OR "bergamot juice"[Title/Abstract] OR "bergamot peel"[Title/Abstract] OR "Bergamot Extract"[Title/Abstract] OR "KoksalGarry"[Title/Abstract] OR "Citrus bergamia Risso et Poiteau"[Title/Abstract] OR "Bergamot Citrus"[Title/Abstract] OR "Bergamot supplementation"[Title/Abstract] OR "Bergamot flavonoids"[Title/Abstract] OR "Citrus Bergamia juice"[Title/Abstract] OR "Citrus Bergamia Extract"[Title/Abstract] | 23,329 |
| #2 | Intervention[Title/Abstract] OR "Intervention Study"[Title/Abstract] OR "Intervention Studies"[Title/Abstract] OR "controlled trial"[Title/Abstract] OR random*[Title/Abstract] OR placebo[Title/Abstract] OR "clinical trial"[Title/Abstract] OR Trial[Title/Abstract] OR "randomized controlled trial"[Title/Abstract] OR "randomized clinical trial"[Title/Abstract] OR RCT[Title/Abstract] OR blinded[Title/Abstract] OR "double blind"[Title/Abstract] OR "double blinded"[Title/Abstract] OR trial*[Title/Abstract] OR "Pragmatic Clinical Trial"[Title/Abstract] OR "Cross-Over Studies"[Title/Abstract] OR "Cross-Over"[Title/Abstract] OR "Cross-Over Study"[Title/Abstract] OR parallel[Title/Abstract] OR "parallel study"[Title/Abstract] OR "parallel trial"[Title/Abstract] OR "Clinical Trial" [Publication Type] OR "Randomized Controlled Trial" [Publication Type] OR "Controlled Clinical Trial" [Publication Type] OR "Random Allocation"[Mesh] OR "Randomized Controlled Trials as Topic"[Mesh] OR "Pragmatic Clinical Trial" [Publication Type] OR "Pragmatic Clinical Trials as Topic"[Mesh] OR "Double-Blind Method"[Mesh] OR "Single-Blind Method"[Mesh] OR "Cross-Over Studies"[Mesh] | 3,820,877 |
| #3 | #1 AND #2 | 2,404 |

**Web of Science**

Number of localized studies: 147

|  | **Descriptors** | **Number of studies reached** |
| --- | --- | --- |
| #1 | TS=("Bergamot" OR "Citrus Bergamia" OR "bergamot essential oil" OR "bergamot juice" OR "bergamot peel" OR "Bergamot Extract" OR "Bergamot Leaves Extract" OR "KoksalGarry" OR "Citrus bergamia Risso et Poiteau" OR "Bergamot Citrus" OR "Citrus bergamias" OR "Bergamot supplementation" OR "Bergamot flavonoids" OR "Citrus Bergamia juice" OR "Citrus Bergamia Extract") | 1,071 |
| #2 | TS=(Intervention OR “Intervention Study” OR “Intervention Studies” OR “controlled trial” OR random* OR placebo OR “clinical trial” OR Trial OR “randomized controlled trial” OR “randomized clinical trial” OR RCT OR blinded OR “double blind” OR “double blinded” OR trial* OR “Pragmatic Clinical Trial” OR “Cross-Over Studies” OR “Cross-Over” OR “Cross-Over Study” OR parallel OR “parallel study” OR “parallel trial”) | 6,536,165 |
| #3 | #1 AND #2 | 147 |

**Scopus**

Number of localized studies: 206

|  | **Descriptors** | **Number of studies reached** |
| --- | --- | --- |
| #1 | TITLE-ABS-KEY ( "Bergamot" OR "Citrus Bergamia" OR "bergamot essential oil" OR "bergamot juice" OR "bergamot peel" OR "Bergamot Extract" OR "Bergamot Leaves Extract" OR "KoksalGarry" OR "Citrus bergamia Risso et Poiteau" OR "Bergamot Citrus" OR "Citrus bergamias" OR "Bergamot supplementation" OR "Bergamot flavonoids" OR "Citrus Bergamia juice" OR "Citrus Bergamia Extract" ) | 1,361 |
| #2 | TITLE-ABS-KEY (Intervention OR "Intervention Study" OR "Intervention Studies" OR "controlled trial" OR random* OR placebo OR "clinical trial" OR trial OR "randomized controlled trial" OR "randomized clinical trial" OR rct OR blinded OR "double blind" OR "double blinded" OR trial* OR "Pragmatic Clinical Trial" OR "Cross-Over Studies" OR "Cross-Over" OR "Cross-Over Study" OR parallel OR "parallel study" OR "parallel trial") | 8,284,103 |
| #3 | #1 AND #2 | 206 |

**Embase**

Number of localized studies: 10,930

|  | **Descriptors** | **Number of studies reached** |
| --- | --- | --- |
| #1 | 'Citrus'/exp OR 'Citrus':ti,ab OR 'Bergamot':ti,ab OR 'Citrus Bergamia':ti,ab OR 'bergamot essential oil':ti,ab OR 'bergamot juice':ti,ab OR 'bergamot peel':ti,ab OR 'Bergamot Extract':ti,ab OR 'Bergamot Leaves Extract':ti,ab OR 'KoksalGarry':ti,ab OR 'Citrus bergamia Risso et Poiteau':ti,ab OR 'Bergamot Citrus':ti,ab OR 'Citrus bergamias':ti,ab OR 'Bergamot supplementation':ti,ab OR 'Bergamot flavonoids':ti,ab OR 'Citrus Bergamia juice':ti,ab OR 'Citrus Bergamia Extract':ti,ab | 29,070 |
| #2 | 'randomized controlled trial'/exp OR 'randomized controlled trial (topic)'/exp OR 'pragmatic trial'/exp OR 'clinical trial'/exp OR 'clinical trial (topic)'/exp OR 'intervention study'/exp OR 'controlled study'/exp OR 'controlled clinical trial'/exp OR 'double blind procedure'/exp OR 'single blind procedure'/exp OR 'crossover procedure'/exp OR 'parallel design'/exp | 12,227,983 |
| #3 | #1 AND #2 | 10,930 |

**Cochrane**

Number of localized studies: 127

Limits: TRIALS

|  | **Descriptors** | **Number of studies reached** |
| --- | --- | --- |
| #1 | ("Bergamot" OR "Citrus Bergamia" OR "bergamot essential oil" OR "bergamot juice" OR "bergamot peel" OR "Bergamot Extract" OR "Bergamot Leaves Extract" OR "KoksalGarry" OR "Citrus bergamia Risso et Poiteau" OR "Bergamot Citrus" OR "Citrus bergamias" OR "Bergamot supplementation" OR "Bergamot flavonoids" OR "Citrus Bergamia juice" OR "Citrus Bergamia Extract"):ti,ab,kw | 127 |

| **Supplementary Table 2.** A summary of excluded articles after full text review | |
| --- | --- |
| **Author, Year (Ref.)** | **Reason** |
| Gliozzi, 2014 (1) | Insufficient data |
| Babish, 2016 (2) | Not interested study design |
| Toth, 2016 (3) | Not interested study design |
| Bruno, 2017 (4) | Not interested study design |
| Nauman, 2019 (5) | Review |

**Supplementary Figure 1.** Random-effects meta-regression plots of the association between bergamot dosage (mg/d) and cardiovascular disease outcomes (a: body mass index, b: waist circumference, C: weight, d: diastolic blood pressure, e: systolic blood pressure, f: glucose, g: homeostatic model assessment of insulin resistance, h: insulin, i: high-density lipoprotein cholesterol, j: low-density lipoprotein cholesterol, k: total cholesterol, m: triglycerides, n: alanine aminotransferase, o: aspartate aminotransferase, p: gamma-glutamyl transferase)

**Supplementary Figure 2.** Forest plot detailing weighted mean difference and 95% confidence intervals (CIs) for the effect of folic acid supplementation on; A) SBP; and B) DBP.

**Supplementary Figure 2.** Forest plot of the effects of chia product supplement on anthropometric measures.

**Supplementary Figure 4.** Forest plot of the effects of chia product supplement on glycemic indices.

**Supplementary Figure 2.** Random-effects meta-regression plots of the association between duration of intervention and cardiovascular disease outcomes (a: body mass index, b: waist circumference, C: weight, d: diastolic blood pressure, e: systolic blood pressure, f: glucose, g: homeostatic model assessment of insulin resistance, h: insulin, i: high-density lipoprotein cholesterol, j: low-density lipoprotein cholesterol, k: total cholesterol, m: triglycerides, n: alanine aminotransferase, o: aspartate aminotransferase, p: gamma-glutamyl transferase)

**Supplementary Figure 3.** Non-linear dose-response relations between bergamot dosage (mg/d) and cardiovascular disease outcome (a: body mass index, b: waist circumference, C: weight, d: diastolic blood pressure, e: systolic blood pressure, f: glucose, g: homeostatic model assessment of insulin resistance, h: insulin, i: high-density lipoprotein cholesterol, j: low-density lipoprotein cholesterol, k: total cholesterol, m: triglycerides, n: alanine aminotransferase, o: aspartate aminotransferase, p: gamma-glutamyl transferase)

**Supplementary Figure 4.** Non-linear dose-response relations between duration of intervention and cardiovascular disease outcomes (a: body mass index, b: waist circumference, C: weight, d: diastolic blood pressure, e: systolic blood pressure, f: glucose, g: homeostatic model assessment of insulin resistance, h: insulin, i: high-density lipoprotein cholesterol, j: low-density lipoprotein cholesterol, k: total cholesterol, m: triglycerides, n: alanine aminotransferase, o: aspartate aminotransferase, p: gamma-glutamyl transferase)

**Supplementary Figure 2.** Forest plot of the effects of chia product supplement on anthropometric measures.

**Supplementary Figure 4.** Forest plot of the effects of chia product supplement on glycemic indices.

# **Supplemental** **References**

1.	Gliozzi M, Carresi C, Musolino V, Palma E, Muscoli C, Vitale C, et al. The effect of bergamot-derived polyphenolic fraction on LDL small dense particles and non alcoholic fatty liver disease in patients with metabolic syndrome. Advances in Biological Chemistry. 2014;4(2):129-37.

2.	Babish JG, Dahlberg CJ, Ou JJ, Keller WJ, Gao W, Kaadige MR, et al. Synergistic in vitro antioxidant activity and observational clinical trial of F105, a phytochemical formulation including Citrus bergamia, in subjects with moderate cardiometabolic risk factors. Canadian journal of physiology and pharmacology. 2016;94(12):1257-66.

3.	Toth PP, Patti AM, Nikolic D, Giglio RV, Castellino G, Biancucci T, et al. Bergamot reduces plasma lipids, atherogenic small dense LDL, and subclinical atherosclerosis in subjects with moderate hypercholesterolemia: a 6 months prospective study. Frontiers in pharmacology. 2016;6:299.

4.	Bruno A, Pandolfo G, Crucitti M, Maisano A, Zoccali RA, Muscatello MRA. Metabolic outcomes of bergamot polyphenolic fraction administration in patients treated with second-generation antipsychotics: A pilot study. The Journal of Nutritional Biochemistry. 2017;40:32-5.

5.	Nauman MC, Johnson JJ. Clinical application of bergamot (Citrus bergamia) for reducing high cholesterol and cardiovascular disease markers. Integrative food, nutrition and metabolism. 2019;6(2):10.15761/IFNM. 1000249.
